# Teloxantron inhibits the processivity of telomerase with preferential DNA damage on telomeres

**DOI:** 10.1038/s41419-022-05443-y

**Published:** 2022-11-28

**Authors:** Natalia Maciejewska, Mateusz Olszewski, Jakub Jurasz, Maciej Baginski, Maryna Stasevych, Viktor Zvarych, Marco Folini, Nadia Zaffaroni

**Affiliations:** 1 Department of Pharmaceutical Technology and Biochemistry, Gdansk University of Technology, Gdansk, Poland; 2 Department of Technology of Biologically Active Substances, Pharmacy and Biotechnology, Lviv Polytechnic National University 13, Lviv, Ukraine; 3 Department of Applied Research and Technological Development, Fondazione IRCCS Istituto Nazionale dei Tumori di Milano, Milan, Italy

**Keywords:** Target identification, Non-small-cell lung cancer, DNA damage response, Pharmaceutics

## Abstract

Telomerase reactivation is one of the hallmarks of cancer, which plays an important role in cellular immortalization and the development and progression of the tumor. Chemical telomerase inhibitors have been shown to trigger replicative senescence and apoptotic cell death both in vitro and in vivo. Due to its upregulation in various cancers, telomerase is considered a potential target in cancer therapy. In this study, we identified potent, small-molecule telomerase inhibitors using a telomerase repeat amplification protocol assay. The results of the assay are the first evidence of telomerase inhibition by anthraquinone derivatives that do not exhibit G-quadruplex-stabilizing properties. The stability of telomerase in the presence of its inhibitor was evaluated under nearly physiological conditions using a cellular thermal shift assay. Our data showed that the compound induced aggregation of the catalytic subunit (hTERT) of human telomerase, and molecular studies confirmed the binding of the hit compound with the active site of the enzyme. The ability of new derivatives to activate DNA double-strand breaks (DSBs) was determined by high-resolution microscopy and flow cytometry in tumor cell lines differing in telomere elongation mechanism. The compounds triggered DSBs in TERT-positive A549 and H460 lung cancer cell lines, but not in TERT-negative NHBE normal human bronchial epithelial and ALT-positive U2OS osteosarcoma cell lines, which indicates that the induction of DSBs was dependent on telomerase inhibition. The observed DNA damage activated DNA damage response pathways involving ATM/Chk2 and ATR/Chk1 cascades. Additionally, the compounds induced apoptotic cell death through extrinsic and intrinsic pathways in lung cancer cells. Taken together, our study demonstrated that anthraquinone derivatives can be further developed into novel telomerase-related anticancer agents.

## Introduction

Telomerase, a ribonucleoprotein complex that adds hexanucleotide sequence to the 3′ end of telomeres, is a promising target for drugs used in anticancer therapy. In normal somatic cells, the critical shortening of telomeres during successive cell divisions results in telomere dysfunctions, eventually leading to replicative senescence and cell growth arrest [1–3]. Most cancers may bypass replicative senescence by activating the telomerase enzyme, which is composed of a reverse transcriptase subunit (TERT), a template containing noncoding RNA (TERC), and accessory proteins such as dyskerin and telomerase Cajal body protein 1 [4]. Additional processive extension of telomeres necessitates the binding of the telomerase enzyme to these sequences through a six-protein complex called shelterin [5, 6].

The activation of telomerase plays a critical role in tumorigenesis. Increased activity of this enzyme is often associated with uncontrolled growth of cells, which is a well-known hallmark of cancer [7]. Telomerase is expressed and activated in 85–90% of cancers. Due to its near universality, high specificity to cancer cells, and ability to confer replicative immortality, telomerase and specifically its catalytic subunit hTERT (human TERT) have become an attractive target for anticancer therapy.

The inhibition of telomerase activity may cause telomere attrition, which in turn may result in cell senescence and/or apoptosis induction [8]. However, even short-term treatment with telomerase inhibitors could induce cell death because of the well-recognized extra-telomeric functions of the enzyme. In fact, apart from the canonical telomere lengthening activity, telomerase has been shown to regulate many physiological processes, including cell growth/proliferation, cell survival, mitochondrial metabolism upon oxidative stress, and modulation of chromatin structure [9]. Additionally, telomerase participates in the transcriptional regulation of gene expression in many signaling pathways, such as Wnt/β-catenin, p65, and NF-κB (nuclear factor kappa-light-chain-enhancer of activated B cells) pathways [10].

There are several approaches to target telomerase, including oligonucleotides or small molecules directly interfering with telomerase, immunotherapy against hTERT tumor-associated antigens, or indirect methods such as targeting telomerase function or regulation by the modulation of hTERT gene expression, the stabilization of telomeric G-quadruplex (G4) structures, or the use of nucleoside analogs [11, 12]. Among small-molecule inhibitors, only non-nucleotidic BIBR1532 has been extensively studied as a telomerase inhibitor, but its clinical applicability is limited due to its poor pharmacokinetic properties [13].

The limited success in the field of small molecule-based telomerase inhibitors has resulted in the search for new compounds by many groups. In this work, we propose the development of a new therapeutic approach that efficiently targets hTERT. Previous studies showed that dithiocarbamates of 9,10-anthracenedione exhibited high cytotoxicity at micromolar and submicromolar concentrations in A549 cells, an in vitro model of non-small cell lung cancer (NSCLC) [14]. However, their exact molecular mechanism of action was unknown. In the present study, we analyzed the antitumor activity of these compounds in cancer cell lines characterized by different telomere elongation mechanisms. We examined their effects in NSCLC cells as well as their ability to induce persistent DNA damage at telomeres causing apoptotic cell death. Our results proved that these molecules can be promising drug candidates for telomerase-positive cancers.

## Results

### 9,10-Anthracenedione derivatives inhibited telomerase activity in a dose-dependent manner

**Fig. 1:**
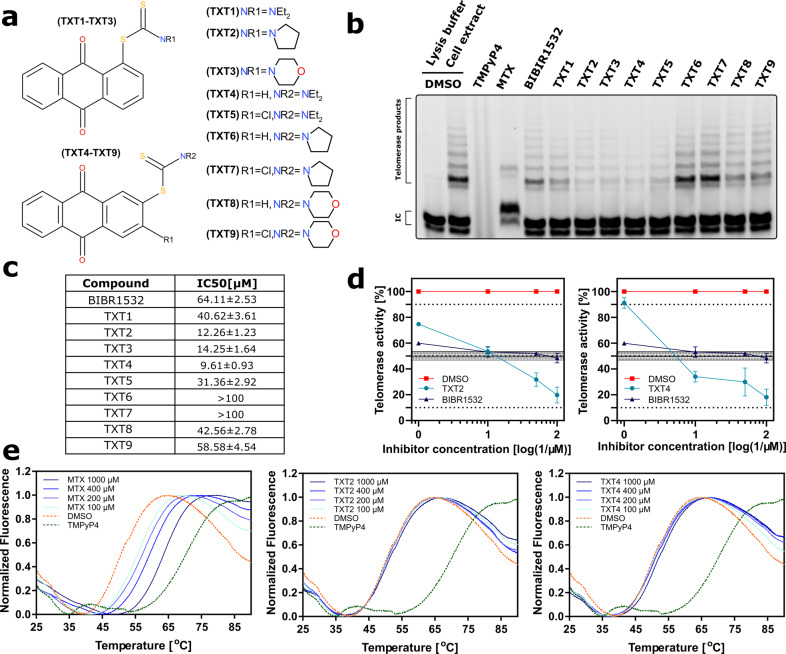
TXT derivatives inhibit telomerase activity. **a** Chemical structure of 9,10-anthracenedione derivatives (TXT). **b** Representative images of the TRAP assay were used to detect telomerase activity in the presence of TXT (100 µM) and reference compounds: TMPyP4 (10 µM), MTX (10 µM), and BIBR1532 (100 µM). IC: internal standard. **c** The table reports the IC_50_ values (μM) of the reference compound BIBR1523 and TXT derivatives for the inhibition of telomerase activity evaluated by test-tube TRAP assay. **d** Quantification of telomerase activity by test-tube TRAP assay in the presence of increasing concentrations of the indicated compounds. Data have been reported as a percentage of telomerase activity with respect to DMSO as a function of compound concentrations and represent mean values ± sd of at least three independent experiments. **e** FRET melting profiles of a human telomeric DNA sequence in the presence of increasing concentrations of MTX, TXT2, or TXT4 compounds. DMSO and TMPyP4 have been used as negative and positive controls for telomeric G4 stabilization, respectively.

The effect of anthraquinone derivatives (Fig. 1a) on telomerase activity was comparatively examined by TRAP assay using the following reference compounds: BIBR1532 (a non-competitive inhibitor that blocks the enzymatic activity of TERT [13]), TMPyP4 (a cationic porphyrin that may indirectly inhibit telomerase activity by stabilizing telomeric G4 [15, 16]) and mitoxantrone MTX (an anthracenedione-based antineoplastic agent belonging to the same chemical family of tested compounds and showing G4 stabilizing properties) [17]. Our results revealed that, except for TXT6 and TXT7 that apparently lack telomerase inhibitory effect at tested concentrations, all TXT compounds showed to inhibit telomerase activity in the test tube assay (Fig. 1b, d), with calculated IC_50_ within the micromolar range (Fig. 1c). In particular, among tested compounds, TXT2, TXT3, and TXT4 (Teloxantron) showed the greater inhibitory effect on telomerase activity (Fig. 1c) with TXT4 showing the lowest calculated IC_50_ value (9.61 ± 0.93 µM), which was about 7-fold lower than that of the reference compound BIBR1532 (64.11 ± 2.53 µM; Fig. 1c). On the other hand, the reference compound TMPyP4 had a negligible inhibitory effect on telomerase activity at low concentrations (0.01 and 0.1 μM; Supplementary Fig. S1). Conversely, it completely inhibited Taq polymerase activity at higher concentrations, as indicated by the lack of internal standard amplification at 1 and 10 μM (Supplementary Fig. S1), thus preventing the proper evaluation of telomerase activity by the TRAP assay. In comparison, none of the TXT compounds inhibited Taq polymerase at concentrations up to 100 µM, as indicated by a consistent signal obtained from the amplification of the internal standard control (Supplementary Fig. S1). Furthermore, MTX showed limited telomerase inhibitory activity in the test-tube TRAP assay (Supplementary Fig. S1), except for a partial inhibition of the enzyme’s activity at the highest concentration, which was however paralleled by a low amplification of the internal standard (Supplementary Fig. S1), thus making impossible to calculate a reliable IC_50_ value and make a proper comparison with TXT compounds.

Several anthraquinone derivatives have been shown to interact with telomeric DNA via G4 stabilization [18, 19]. Therefore, we evaluated the ability of our compounds to promote telomeric G4 DNA thermal stabilization using FRET melting assay in comparison to the reference compound TMPyP4 and MTX known for having G4 stabilizing properties. In particular, FRET melting profiles indicated that both reference compounds showed good telomeric G4 stabilizing properties, with ΔTm values of +15.8 and +19.7 °C for MTX and TMPyP4, respectively (Fig. 1e and Supplementary Fig. S2). Conversely, none of our tested compounds showed marked telomeric G4 stabilizing properties, as revealed by the lack of a significant shift of the Tm and the melting profiles that were superimposable to that observed for DMSO (Fig. 1e). This evidence would indicate that the 9,10-anthraquinone derivative-mediated inhibition of telomerase activity observed in the TRAP assay occurred with a mechanism that does not involve telomeric G4 stabilization.

### Evaluation of TXT as a telomerase inhibitor using molecular docking analysis

**Fig. 2:**
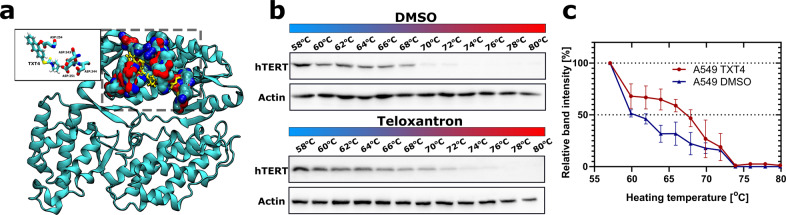
TXT4 interacts with TERT and favors its aggregation. **a** Representative image of the structure of telomerase together with the TXT4 ligand. The figure shows the whole structure of telomerase and the active site pocket in which the ligand and aspartic acid (both in yellow) fit. The inset shows the positioning of the ligand relative to D251, D254, D343, and D344. **b** Representative western blots showing the thermal stabilization of TERT protein upon treatment with Teloxantron (TXT4) with respect to DMSO. **c** The band intensity of hTERT in DMSO- and TXT4-treated A549 cells has been reported as a function of heating temperatures. Data have been reported as the percentage of the band intensity with respect to the samples exposed to the lowest temperature (58 °C) upon densitometric analyses of western immunoblots and represent mean values ± sd from at least three independent experiments. The arrow indicates the shift towards the right of the melting curve, and the consequent increase in ∆Tm indicates the capability of a compound to interfere with the folding of the target protein.

On the basis of FRET data showing that TXT compounds are devoid of G4 stabilizing properties, we focused on TXT4 (Teloxantron, the most potent inhibitor of telomerase activity; Fig. 1c) and investigated its possible mechanism of action by evaluating its interaction with hTERT using in silico molecular docking analyses. Docking poses and binding energies were compared with pterostilbene, a compound that interacts with the active site of the enzyme [20]. To estimate the ligand-receptor affinity, an empirical scoring function inspired by the X-score function was used in Vina [21]. The simulations of both dockings were narrowed down to 10 results based on the score function, of which, after careful visual inspection, the best ones were selected taking into account their spatial arrangement and docking score. The docking results indicated that TXT4 showed comparable binding energies (on average it showed a better result than pterostilbene by 0.6 kcal/mol) and similar docking poses as the reference compound, which suggests that it may have a similar affinity. As shown in Fig. 2a, TXT4 fitted perfectly in the pocket of the active site and formed active hydrogen bonds with D343 and D251. D344 is slightly distant; however, the protein was stiff when docked, and visual inspection showed a lack of ligand space. When running an equilibrium simulation, this ligand may go deeper, which is a good starting point for further research. In addition, one more aspartic acid residue, D254, was noticed, which in most of the simulations was in closer contact with the ligand forming bonds. Based on molecular docking simulations alone, it is impossible to conclude whether the amino acids we propose form bonds with the ligand and favor its affinity for the active site.

In addition, the ability of TXT4 to interact with and likely stabilize telomerase in intact cells was analyzed by the cellular thermal shift assay (CETSA) (Fig. 2b, c). CETSA allows rapid assessment of target engagement of drugs under a physiologically relevant condition based on the ligand-induced thermal stabilization of the target protein. In particular, our data showed that a prominent shift in the melting curve of hTERT and a concomitant increase in the denaturation temperature (ΔTm = 10.23 °C) occurred in the presence of TXT4 compared to DMSO. This evidence suggests that the binding of TXT4 to native hTERT protein would induce its aggregation in living systems.

### The exposure of TERT-positive cells to TXT compounds elicits DNA damage

**Fig. 3:**
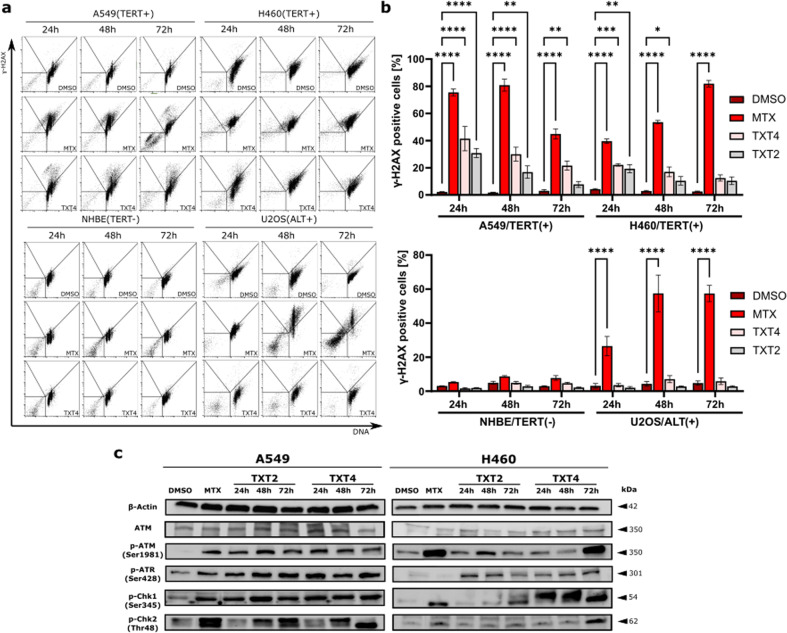
TXT derivatives evoke a DNA damage response in telomerase-positive cancer cells. **a** Representative histograms of the time-dependent assessment of γ-H2AX accumulation by flow cytometry in A549, H460, NHBE, and U2OS cell lines exposed to equitoxic amounts of TXT2, TXT4, and of reference compound MTX. **b** Percentage of cells that stained positive for γ-H2AX as assessed by flow cytometry. Data represent mean values ± sd from at least three independent experiments. *p < 0.01, **p < 0.001, ***p < 0.0001, and ****p < 0.00001 (two-way ANOVA and post hoc Dunnett’s test). **c** Representative Western immunoblot showing the levels of DNA damage-related proteins in TXT2- and TXT4- treated A549 and H460 cells. DMSO and MTX were included as negative and positive controls, respectively. β-actin was used to ensure equal protein loading.

Since a link between telomerase inhibition and DNA damage has been documented [22], the extent of DNA damage induction was comparatively investigated in TERT-positive (A549 and H460) and ALT-positive (U2OS) cancer cells as well as in hTERT-negative normal human bronchial epithelial (NHBE) cells exposed to equitoxic concentrations of tested compounds. In particular, the exposure of A549 and H460 cells to TXT2 and TXT4 resulted in a remarkable and time-dependent induction of DNA damage, as assessed by flow cytometric analysis of γ-H2AXstaining, though to a lesser extent with respect to the levels of damage observed upon exposure to the reference compound MTX (Fig. 3a, b). On the other hand, TXT2 and TXT4 failed to induce DNA damage in the ALT-positive osteosarcoma U2OS cells, which in turn were characterized by a remarkable accumulation of γ-H2AX upon exposure to MTX (Fig. 3a, b), whereas equitoxic amounts of all compounds failed to elicit DNA damage in TERT-negative NHBE cells (Fig. 3a, b).

In addition, the assessment of the DDR protein network by Western immunoblot showed that TXT2 and TXT4 caused an increase in the phosphorylation of ATM and ATR, two main DDR signaling kinases [23], in both A549 and H460 cells, though with a greater effect in A549 compared to H460 cells. Additionally, phosphorylation of Chk2 at threonine 68 and of Chk1 at serine 345, which are downstream targets of ATM and ATR, respectively, was induced by both compounds in TERT-positive NSCLC cells (Fig. 3c). Conversely, the exposure of ALT-positive cancer cells to TXT compounds failed to evoke the activation of both ATM- and ATR-dependent signaling cascade (Supplementary Fig. S3).

**Fig. 4:**
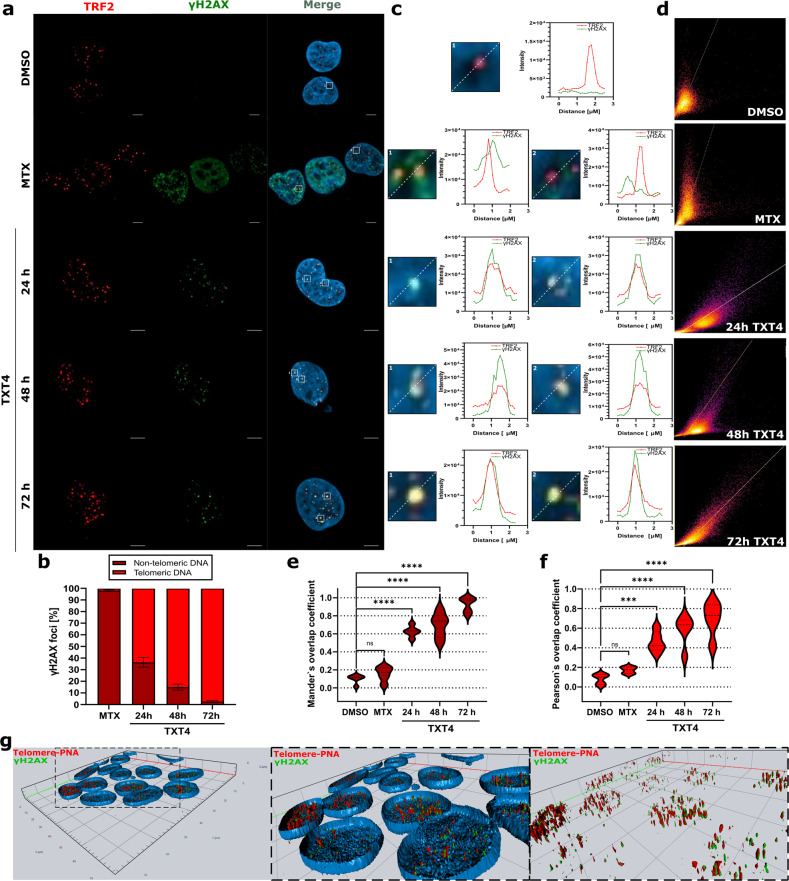
TXT4 induces telomere dysfunctions in TERT-positive cells. **a** Representative high-resolution images obtained by laser scanning confocal microscopy showing the colocalization of γ-H2AX and TRF2 as a function of time in A549 cells after treatment with TXT4. DMSO and MTX were included as reference controls. Scale bars=10 μm. Nuclei were counterstained with DAPI; **b** Quantification of γ-H2AX nuclear foci. Bright red bars indicate the fraction (%) of γ-H2AX nuclear foci that localize at the telomeric level within the overall amount of DNA damage nuclear foci; **c** Representative line scans showing the overlapping between γ-H2AX (green) and TRF2 (red) fluorescent signal corresponding to the marked area reported in **a**; **d** Scatter plots showing the overlapping between γ-H2AX (green) and TRF2 (red) fluorescent signal corresponding to the colocalization of pixels in the images reported in **a**; **e**, **f** Graphical representation of the calculated Mander’s and Pearson’s overlapping coefficients. Error bars represent the sd of data obtained in at least *n* = 15 randomly selected locations on the slide. ns > 0.05, **p < 0.001, ***p < 0.0001, and ****p < 0.00001 compared to DMSO (two-way ANOVA); **g** Combined immunofluorescence and telomeric FISH (fluorescence in situ hybridization) showing the co-localization between γ-H2AX (green) and telomeric DNA (red) in A549 cells exposed for 72 h to TXT4. A 3-D reconstruction of microscopy images captured in Z-stack has been reported.

To investigate the possible influence of TXT-mediated inhibition of telomerase activity on the induction of telomere dysfunction-induced foci (TIFs), which are markers for telomeric localized DNA damage, the co-localization of γ-H2AX and of the telomeric repeat binding factor 2 (TRF2, one of the shelterin-associated proteins that can directly bind to telomeric DNA) was investigated by immunofluorescence microscopy. In particular, a remarkable and time-dependent increase in telomere dysfunctions (i.e., TIFs) was appreciable in Teloxantron- with respect to DMSO- or MTX-treated A549 cells (Fig. 4a-b and Supplementary Figs. S4-S5), as indicated by the extent of the overlapping between γ-H2AX and TRF2 fluorescent signal (Fig. 4c, d) as well as by the calculated Mander’s (MCC: 0.922 ± 0.055) and Pearson’s (PCC: 0.723 ± 0.046) correlation coefficients (Fig. 4e, f). This evidence was further confirmed by combined immunofluorescence and telomeric FISH showing that the majority of telomeric signals colocalized with γ-H2AX in A549 cells after 72 h of treatment with TXT4 (Fig. 4g). Conversely, no differences in TIFs formation were observed in NHBE or U2OS cells treated with TXT4 with respect to DMSO (Fig. S6).

Altogether, these findings indicate that the inhibition of hTERT by TXT4 leads to DNA damage, that mainly localizes to telomeric regions.

### TXT stimulated apoptotic cell death via the intrinsic and extrinsic pathway

**Fig. 5:**
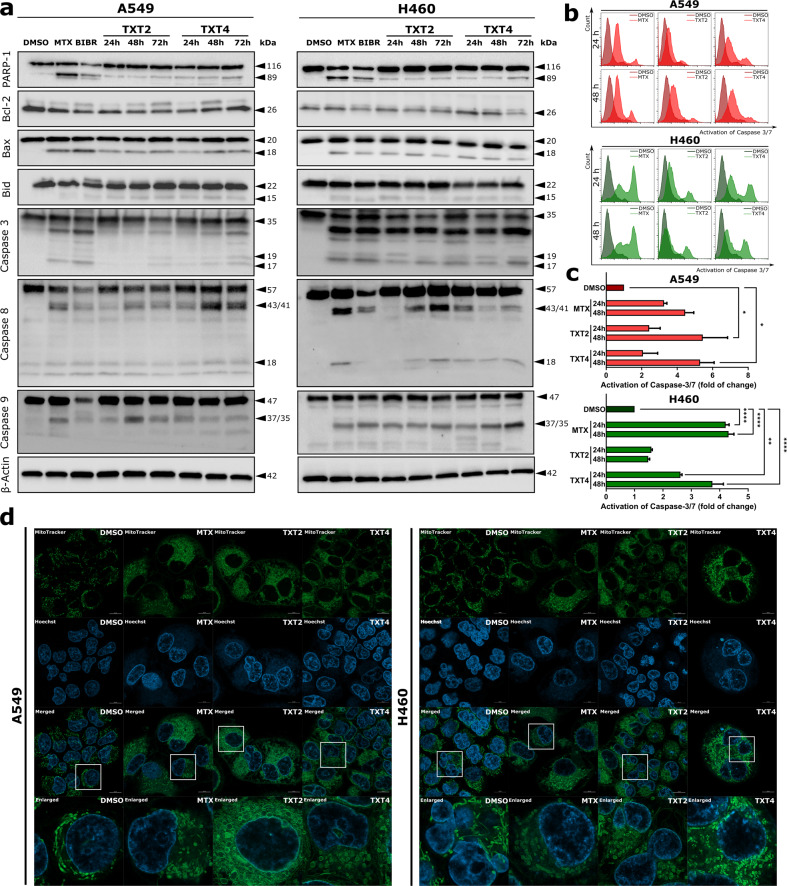
The exposure of TERT-positive cells to TXT compounds may lead to programmed cell death. **a** Representative western immunoblotting showing the amounts of apoptotic-related proteins in A549 and H460 cell lines exposed to TXT compounds. DMSO and MTX were used as negative and positive controls, respectively. β-actin was used to ensure equal protein loading. Numbers on the right indicate the molecular weight (kDa); **b** Representative histograms of flow cytometric analyses of caspase-3/7 activation in A549 and H460 cell lines at 24 and 48 h of treatment with TXT compounds. DMSO and MTX were used as negative and positive controls, respectively; **c** Quantitation of caspase-3/7 activation as assessed by flow cytometry. Data have been reported as fold-change of caspases activation with respect to DMSO-treated cells and represent mean values ± s.d. from at least three independent experiments. **p < 0.001, and ****p < 0.00001 (one-way ANOVA and post hoc Dunnett’s test); **d** Representative images of MitoTracker staining assessed by fluorescence microscopy show the structure of mitochondria in A549 and H460 cells after a 24-h exposure to TXT2 and TXT4. Scale bars = 10 µM. Nuclei were counterstained with DAPI.

To examine whether TXT-mediated DNA damage induction eventually leads to cell death, the possible occurrence of apoptosis was investigated in NSCLC and U2OS cells exposed to equitoxic concentrations of TXT compounds (Supplementary Figs. S7-S10 and Fig. 5). In particular, after 6 h of treatment of A549 cells, the percentage of surviving cells (Annexin V-FITC(-)/7-AAD( − )) slightly decreased after exposure to both compounds (Supplementary Fig. S7). After 24 h of exposure to TXT4, a threefold increase in both early (Annexin V-FITC( + )/7-AAD( − )) and late (Annexin V-FITC( + )/7-AAD( + )) apoptotic fractions was observed in treated cells in comparison to the vehicle, whereas TXT2 induced apoptosis to a smaller extent (Supplementary Fig. S7). Further incubation (48 h) of A549 cells led to a significant decrease in the fraction of live cells, and a concomitant increase in Annexin V-positive cells, confirming phosphatidylserine externalization, and the ongoing apoptotic cell death (Supplementary Fig. S7). Importantly, TXT compounds exhibited more potent proapoptotic properties on A549 cells than both BIBR1532 and MTX reference compounds. Moreover, H460 cells also undergo time-dependent apoptotic cell death after treatment with TXT2 and TXT4, leading to an increase in apoptotic fraction up to 19.11 ± 2.31%, and 27.65 ± 2.89%, respectively (Supplementary Fig. S8). On the other hand, none of the TXT compounds didn’t evoke apoptosis in ALT-positive cells, similar to BIBR1532 (Supplementary Fig. S9). Under the same conditions, a constant number of necrotic fractions (Annexin V(-)/7-AAD( + )) was observed for all cell lines (Supplementary Figs. S7-S9). These results confirmed that TXT2 and TXT4 may trigger apoptotic rather than necrotic cell death in NSCLC cell lines.

Moreover, western immunoblotting analyses of apoptotic-related factors revealed a marked increase in the proteolytic cleavage of PARP-1 in both cell lines exposed to TXT compounds. In addition, a time-dependent decrease in the levels of the inactive (full-length) forms of caspase-8 and caspase-9 and a concomitant appearance of their cleaved forms were also appreciable (Fig. 5a). In parallel, events downstream of caspase-8 activation, such as processing of Bid to its active form tBid/p15, along with the appearance of cleaved forms of effector caspase-3 were also detected by western immunoblotting (Fig. 5a). Of note, TXT-mediated activation of effector caspase-3 and -7 was also confirmed by flow cytometric analysis in both TERT-positive, but not ALT-positive cell lines (Fig. 5b, c, and Supplementary Fig. S10).

Moreover, TXT compounds did not cause detectable changes in the levels of the anti-apoptotic factor Bcl-2 in both cell lines (Fig. 5a) whereas the occurrence of proteolytic cleavage to p18 form of Bax protein, an event known to contribute to mitochondrial dysfunction [24], was appreciable in TERT positive cells upon exposure to TXT compounds (Fig. 5a). In comparison, no changes in the expression of apoptotic-related proteins were observed after treatment of ALT-positive cells with TXT compounds (Supplementary Fig. S10). Fluorescence microscopy analysis of MitoTracker staining was instrumental to delineate the activation of the mitochondrial apoptosis pathway by TXT compounds in more detail. After 24-h treatment with our derivatives, both cell lines showed mitochondria displaying increased fission, revealed as a circular-shaped and short structure (Fig. 5d and Supplementary Fig. S11), which was paralleled by the release of mitochondrial DNA into the cytoplasm, thus indicating the loss of the mitochondrial membrane potential (MMP) that may lead to programmed cell death [25]. Indeed, as shown in Supplementary Fig. S12, both compounds induced depolarization of the mitochondrial membrane in TERT-positive cells, which was revealed as an increase in JC-1 monomers fluorescence. Importantly, neither of the TXT compounds altered MMP in ALT-positive cells (Supplementary Fig. S12).

## Discussion

In this study, we developed highly potent and effective hTERT inhibitors with an anthraquinone core. Anthraquinones have been recognized as cancer growth inhibitors targeting topoisomerases, telomerase, matrix metalloproteinases, and protein kinase [26, 27]. Several drugs containing an anthraquinone scaffold, such as doxorubicin, epirubicin, pixantrone, valrubicin, and MTX, are clinically used to treat various types of cancers [26]. All known telomerase-targeting anthraquinones act as stabilizers of telomeric G4 DNA, altering the binding of telomerase enzyme to DNA and blocking telomerase activity in vitro and vivo [11, 28]. Most of the telomerase-targeting compounds are nonselective because G4 structures also occur in additional G-rich DNA sequences other than telomeres, which may cause high toxicity to normal cells [29]. A major finding of our study is that the tested compounds unexpectedly failed to stabilize telomeric G4, but rather blocked the catalytic activity of TERT protein component. In fact, even if a more advanced in silico molecular modeling methodology (thermodynamics study) may be used to further support our hypothesis on the binding site, in the present study we showed that Teloxantron is able to bind to the active site of TERT and, upon binding, to likely cause protein aggregation in living cells as assessed by CETSA analyses.

Telomerase inhibition affects the normal kinetics of telomere capping, resulting in the accumulation of DNA damage signals. Uncapped telomeres are perceived as DNA damage in the absence of proper capping [30]. However, DNA damage is a double-edged sword guarding the genome. If left unrepaired, DNA damage in a normal cell contributes to mutations, and subsequently genomic instability, which is the major hallmark of cancer development and progression. Nonetheless, agents that are commonly used in cancer treatment, such as platinum drugs, anthracyclines, topoisomerase poisons, and radiation, activate DNA damage in tumor cells causing altered expression of DNA repair genes [31]. In some types of cancers, multiple molecular mechanisms mending DNA lesions at nontelomeric chromosomal sequences are observed, which effectively increase intrinsic resistance to radiotherapy and chemotherapy [32]. DNA on telomeres has a different DNA repair capacity than the rest of the genome, due to the inhibition of the nonhomologous end-joining (NHEJ) repair pathway to prevent end-to-end chromosome fusions. NHEJ is the predominant DSB repair pathway, which is inhibited by shelterin proteins, which block the access to telomeric overhang by promoting the formation of the t-loop. For this reason, telomeric DNA damage is irreparable and leads to persistent DDR which triggers signaling cascades that drive cells to either apoptosis or senescence [29, 33].

Our study revealed a functional link between DNA damage and inhibition of telomerase in cancer cells. Compounds TXT2 and TXT4 activated DSBs in TERT-positive A-549 and H460 cell lines, which was manifested by increased levels of γ-H2AX. On the other hand, none of the tested compounds induced DSBs in TERT-negative normal bronchial epithelial cells or ALT-positive osteosarcoma cells, suggesting that the induction of DSBs is dependent on telomerase inhibition. Further analyses of A549-treated cells showed that TXT4 induced DSBs on telomeres which may affect telomere-maintenance kinetics and homeostasis. In addition, even after 24 h of treatment, a high amount of DNA damage was found in these cells, with a significant proportion in extratelomeric regions. Longer exposure resulted in a marked decrease in DSBs within a chromosome, but without repairing telomeric DNA. It could be hypothesized that the superior distance between broken DNA ends in telomeric regions compared to that of broken ends forming on internal chromosome regions would impede joining, and thus slow down DNA repair in telomeric regions [34]. Moreover, at a high dose, TXT4 could not only suppress the catalytic activity of telomerase but also severely compromise the functions of other proteins involved in the enzyme assembly resulting in telomere damage rather than replicative stress. Short-term exposure to TXT2/TXT4 induced both ATM- and ATR-mediated DDR related to telomere dysfunctions [35]. Activation of ATM, as well as ATR, was followed by the induction of signaling cascade via phosphorylation of its downstream checkpoint effector kinases, Chk1 and Chk2, which are needed for cells to enter mitosis. In addition, telomerase inhibition could cause perturbation of individual subunits from the shelterin protein by compacting telomeric chromatin leads to direct damage to the telomere structure [36]. Indeed, Karlseder et al. described that DNA damage and cell cycle arrest occur as a result of changes in telomerase composition rather than telomere shortening [37]. However, although Nakamura et al. reported that γ-H2AX can be a biomarker to evaluate the efficacy of telomerase inhibition, we cannot exclude that the DSBs observed on telomeres are not only associated with telomerase but may also be related to an additional mechanism leading to cell death [38].

Cells with DNA damage that fail to undergo repair evoke secondary responses, leading to apoptotic cell death through either the mitochondrial (intrinsic) or death receptor (extrinsic) pathway. During the intrinsic-mediated pathway, mitochondrial outer membrane permeabilization (MOMP), which is considered a “point of no return,” directly induces the release of death-promoting proteins [39]. MOMP requires transient exposure of the Bak or Bax BH3 domain and facilitates the efflux of cytochrome c into the cytoplasm, which together with apoptotic protease activating factor 1 activates the initiator caspase 9 [39]. Activated caspase-9 cleaves and activates the downstream effectors caspase-3 and caspase-7. Extrusion of the mitochondrial inner membrane into the cytosol and its permeabilization cause the widening of Bax/Bak pores and trigger the release of mitochondrial DNA [40]. Our study demonstrated that TXT2 and TXT4 triggered apoptotic cell death in A549 and H460 cancer cell lines in a time-dependent manner. Apoptosis was induced by direct activation of caspase-3 or by cleavage of Bid, resulting in mitochondrial dysfunction and subsequent activation of caspase-9 and caspase-3. Additionally, we observed the cleavage of caspase-8, which is an executing protein in the extrinsic apoptotic pathway. In the death receptor pathway, activation of caspase-8 mediates the generation of truncated Bid, forming a signaling link between the extrinsic tumor necrosis factor family and mitochondrial-based apoptotic pathway [41]. The “BH3 domain only” of tBid binds the Bax canonical grove, leading to its oligomerization, as well as MOMP [42]. Treatment with TXT2 and TXT4 led to the activation of both Bid and Bax, which indicates the activation of extrinsic apoptosis involving transmembrane receptor-mediated interactions. Our observations are supported by an earlier study showing that inhibition of telomerase induced both intrinsic and extrinsic apoptosis in acute myeloid leukemia stem cells [43]. Shammas and co-workers reported that siRNA-mediated inhibition of telomerase activated both mitochondrial- and death-receptor-mediated pathways in Barrett’s adenocarcinoma SEG-1 cells [44]. In addition, Giunco et al. observed that short-term inhibition of TERT by BIBR1532 in human malignant B cells xenografted in zebrafish led to apoptosis with induction of DDR [45].

The development of telomerase inhibitors, in general, raises several questions: (i) some studies have shown that the ALT (alternative lengthening of telomere) mechanism may be activated as an adaptive response to telomerase inhibition leading to chemoresistance; (ii) clinical relevance of telomerase inhibition in cancer patients is controversial due to the delayed therapeutic effect (i.e., the lag time needed for telomeres to shorten to a critical size before a cancer cell undergoes senescence and/or cell death) [46, 47]. Nevertheless, evidence suggests that it is beneficial to use this approach in combination therapy with other drugs, such as tyrosine kinase or topoisomerase inhibitors [48, 49]. A recent study by Hu and co-workers showed that treatment of myelofibrosis hematopoietic stem cells and progenitor cells with ruxolitinib, a Janus kinase inhibitor, in combination with imetelstat was more effective than treatment with the kinase inhibitor used as single agent [50]. Similarly, Gupta et al. showed that combined inhibition of telomerase and p21(^waf1^) synergistically suppressed tumor growth in different cell lines [51]. Moreover, Goldblatt, et al. demonstrated that the use of imetelstat combined with paclitaxel, an inhibitor of microtubule depolymerization, caused a significant reduction in the invasive potential of MDA-MB-231 breast cancer cells, allowing for effective growth inhibition at lower doses of both drugs, compared to treatment with the single agents [52]. Of note, such a combination regimen has been evaluated in a phase II clinical trial in patients with locally recurrent or metastatic breast cancer (ClinicalTrials.gov identifier NCT01256762) [53].

In summary, we have presented novel telomerase inhibitors with promising anticancer activity. Further studies are warranted to evaluate the efficacy of TXT4 in targeting cancer cells, including long-term treatment with noncytotoxic concentrations to investigate its effect on telomere shortening and senescence. Although small-molecule inhibitors may effectively target telomerase, cells may possibly gain resistance to telomerase inhibitors after excessive telomere shortening. However, it also implies that there will be a constant demand for innovative telomerase inhibitors/modulators with a unique mechanism of action and the proposed molecules may allow fulfilling this need.

## Materials and methods

### Compounds

TXT compounds were synthesized accordingly to the protocol described earlier [14]. BIBR1532 was purchased from Selleck Chemicals LLC (Houston, TX, USA). MTX was purchased from Sigma-Aldrich (St. Louis, MO, USA). TMPyP4 was purchased from Cayman Chemical (Ann Arbor, MI, USA).

### CETSA

CETSA was performed as described by Jafari et al. [54]. Briefly, cells were cultured in 100 × 20 mm tissue culture dishes until they reached 80% confluence, and treated with 1% (v/v) DMSO (Sigma-Aldrich) or 15 µM TXT4 for 6 h. After exposure to the compound, cells were harvested by scraping, collected by centrifugation at 20,000 × *g* for 10 min at 4 °C, and then resuspended in phosphate-buffered saline (PBS; Sigma-Aldrich). The cell suspension was aliquoted in PCR tubes and heated in a thermal cycler for 3 min at 57–80 °C. Subsequently, cells were lysed by repeated freeze-thaw cycles using liquid nitrogen and a heating block set at 25 °C. The soluble protein fraction was separated by centrifugation at 20,000 × *g* for 20 min at 4 °C and subjected to Western blot analysis. Full and uncropped western blots are presented in Supplemental File.

### Immunofluorescence

Cells (2 × 10^5^) were seeded onto tissue culture plates with a glass slide and allowed to attach overnight. On the next day, cells were exposed to the tested compounds at IC_90_ concentration for the indicated time. Then, cells were washed in PBS, fixed for 15 min at room temperature (RT) with 4% paraformaldehyde (PFA; Sigma-Aldrich) in PBS, and permeabilized for 15 min in 0.25% Triton X-100 (Thermo Fisher Scientific, Waltham, MA, USA) in PBS. Subsequently, cells were blocked with 3% bovine serum albumin (BSA) in PBS for 1 h at RT and incubated with Alexa Fluor 488-conjugated mouse anti-H2AX (pS139) antibody (#560445, 1:200 dilution; BD Pharmingen, San Diego, CA, USA) and/or antirabbit rabbit TRF2 (ab108997, 1:200 dilution; Abcam, Cambridge, UK), or antitelomerase (ab32020, 1:300 dilution; Abcam) for 1.5 h at 37 °C in a humidified chamber. Cells were washed three times with PBS-Tween 20 (PBS-T) and incubated with Alexa Fluor 594-conjugated anti-rabbit IgG antibody (#sc-516250, 1:500 dilution; Santa Cruz Biotechnology, Santa Cruz, CA, USA) for 1 h in a humidified chamber at 37 °C. Cells were washed three times for 10 min with PBS-T, stained with 0.25 µg/ml 4′,6-diamidino-2-phenylindole (DAPI; Sigma-Aldrich) for 15 min, and mounted onto slides with PBS-glycerol (90%) containing 2.5% (w/v) 4-diazobicyclo-(2,2,2-octane) (DABCO; Sigma-Aldrich). The coverslips were washed twice with PBS-T and stained with 0.25 µg/ml DAPI. Images were acquired with an LSM 800 inverted laser scanning confocal microscope (Carl Zeiss; Dresden, Germany) equipped with an Airyscan detector using a ×63 1.4 NA Plan Apochromat objective (Carl Zeiss). The parameters of laser intensity, exposure times, gain settings, and so on were kept constant for both compound-treated and DMSO-treated cells. Correlations of overlapping pixel intensities were calculated using thresholded MCC and PCC from Z-stack images. Data were obtained in at least *n* = 15 randomly selected locations on the slide. The scatter plots and coefficients were analyzed using the JACoP plugin under ImageJ 1.53n software (National Institutes of Health, Bethesda, USA), as previously described [55].

### Combined immunofluorescence and telomere FISH

After immunofluorescence, cells were fixed with 4% PFA in PBS for 15 min and washed twice with PBS. FISH was performed as described by Cesare et al. [56]. Briefly, coverslips were dehydrated with a series of ethanol solutions, denatured for 5 min at 80 °C, and hybridized with Alexa Fluor 647-labeled CCCTAA PNA probe (Panagene Inc, Daejeon, South Korea) overnight at RT. Then, coverslips were washed, dehydrated, mounted onto slides with PBS-glycerol (90%) containing 2.5% (w/v) DABCO, and stained with 0.25 µg/ml DAPI. Cells were visualized and analyzed as described for immunofluorescence.

Other materials and methods used in this study are listed in the Materials and Methods section in the Supplementary information.

## Supplementary information

Reproducibility checklist

Supplemental figures

Supplemental materials and methods

Full-lenght gels and blots

## Data Availability

The datasets presented in the current study are available from the corresponding author upon reasonable request.
