# Supplementary material for: Teloxantron inhibits the processivity of telomerase with preferential DNA damage on telomeres
Source: Cell Death Dis. 2022 Nov 28;13(11):1005. doi: 10.1038/s41419-022-05443-y (PMC9701690; doi:10.1038/s41419-022-05443-y)
Supplement: Supplementary file 2 — Supplemental figures [file 41419_2022_5443_MOESM2_ESM.docx]

**
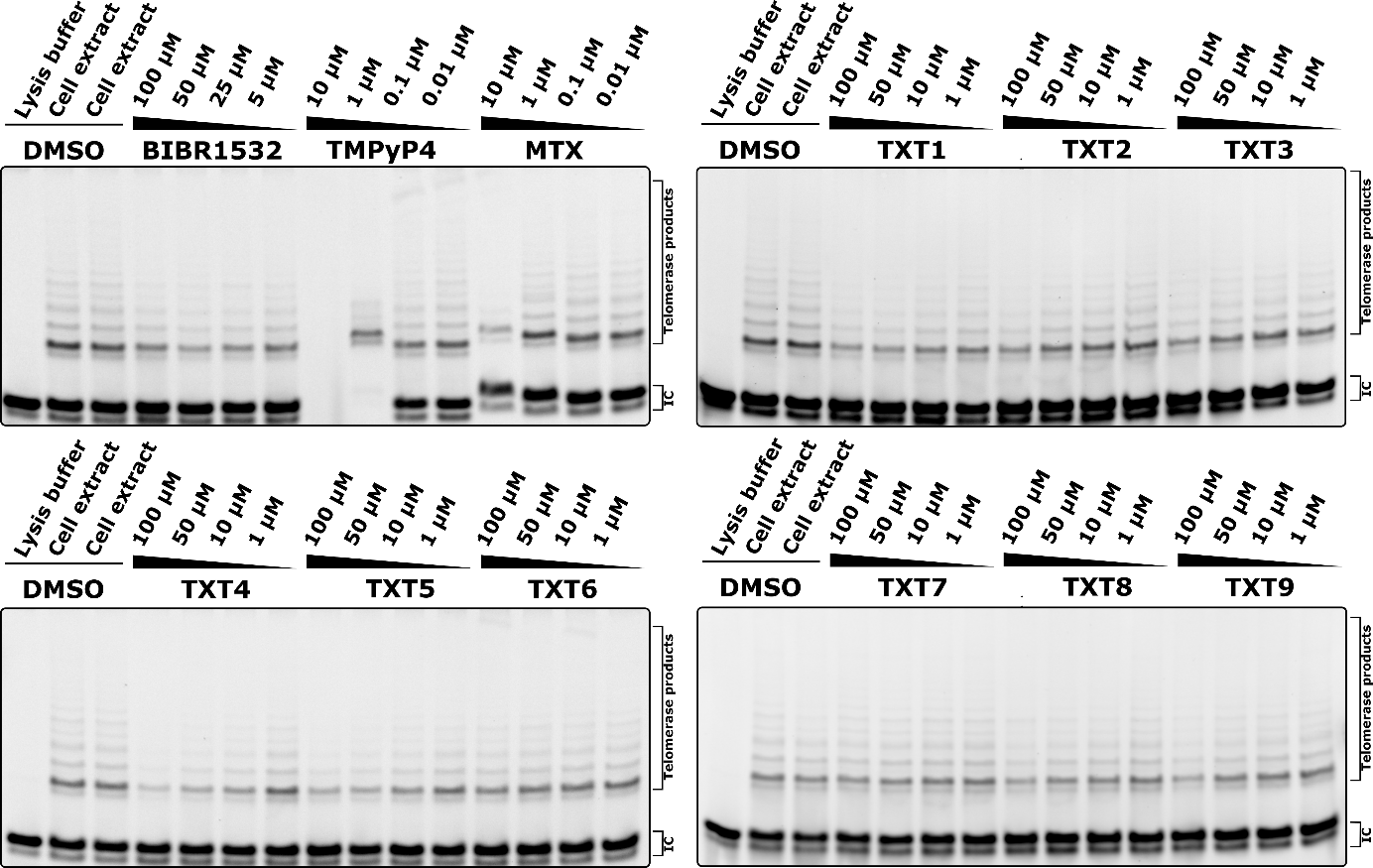
**

**Figure S1** Representative images of the TRAP assay used to detect telomerase activity in the presence of increasing concentrations of TXT and reference compounds. IC: internal standard.

**
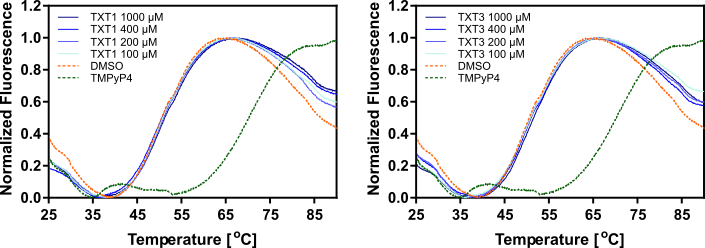
Figure S2** FRET melting profiles of a human telomeric DNA sequence in the presence of increasing concentrations of MTX, TXT2, or TXT4 compounds. DMSO and TMPyP4 have been used as negative and positive controls for telomeric G4 stabilization, respectively.

**
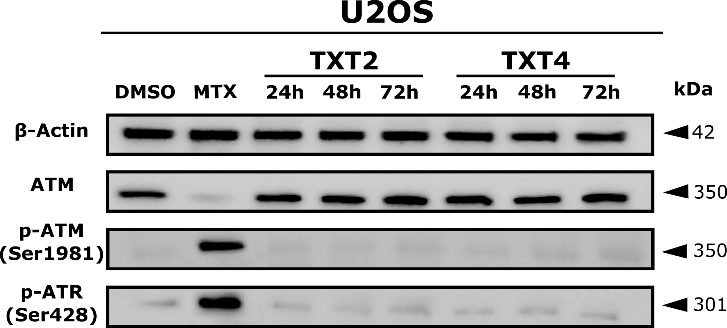
**

**Figure S3** Representative Western immunoblot showing the levels of DNA damage-related proteins in TXT2- and TXT4- treated U2OS cells. DMSO and MTX were included as negative and positive controls, respectively. β-actin was used to ensure equal protein loading.


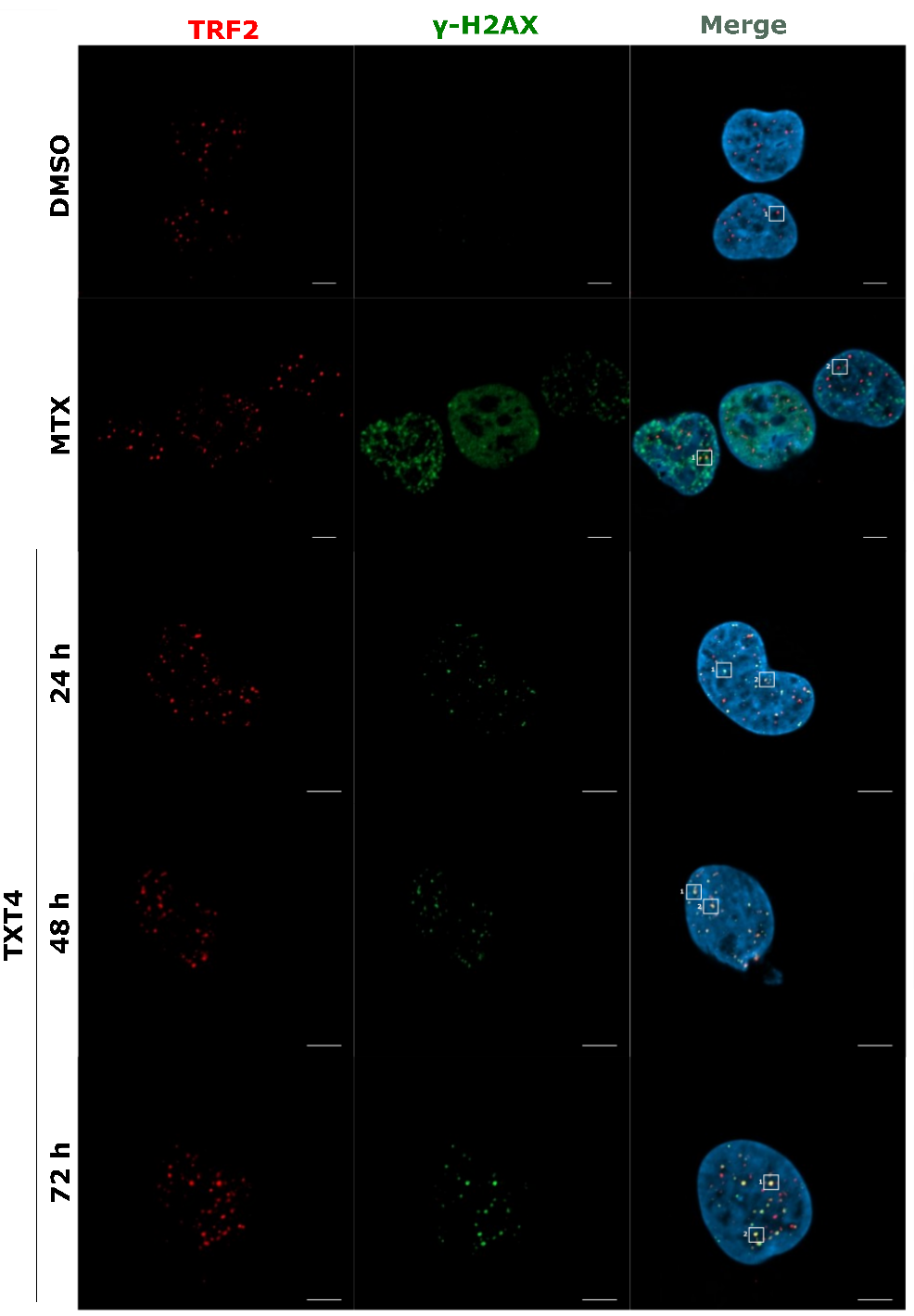


**Figure S4** Original images those presented in Figure 4a of the main article.


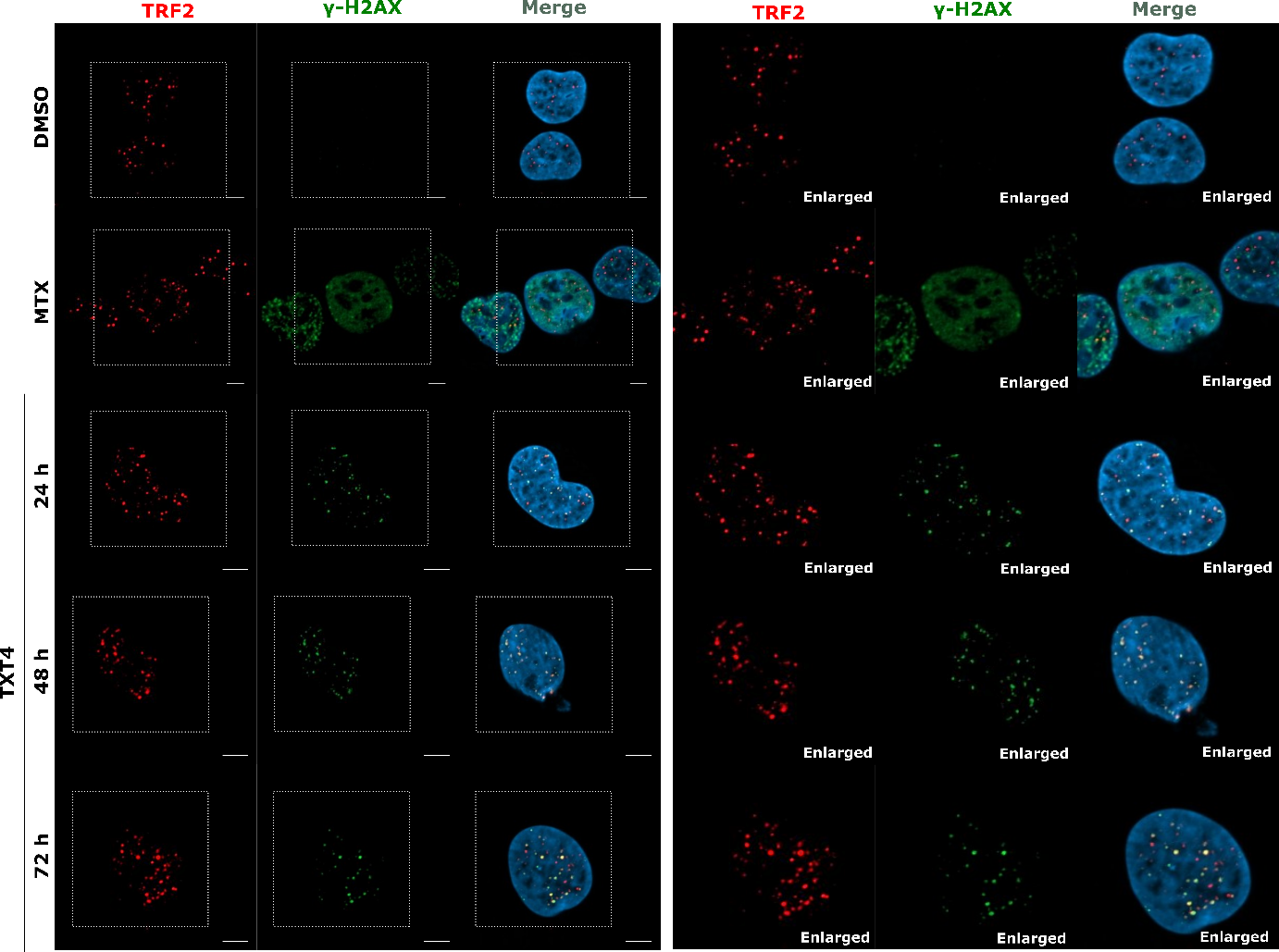


**Figure S5** Enlarged microscopy images show the colocalization of γ-H2AX and TRF2 in A549 cells after treatment with TXT4, corresponding to the marked area reported on the left panel. DMSO and MTX were included as reference controls. Scale bars=10 μm. Nuclei were counterstained with DAPI.

**
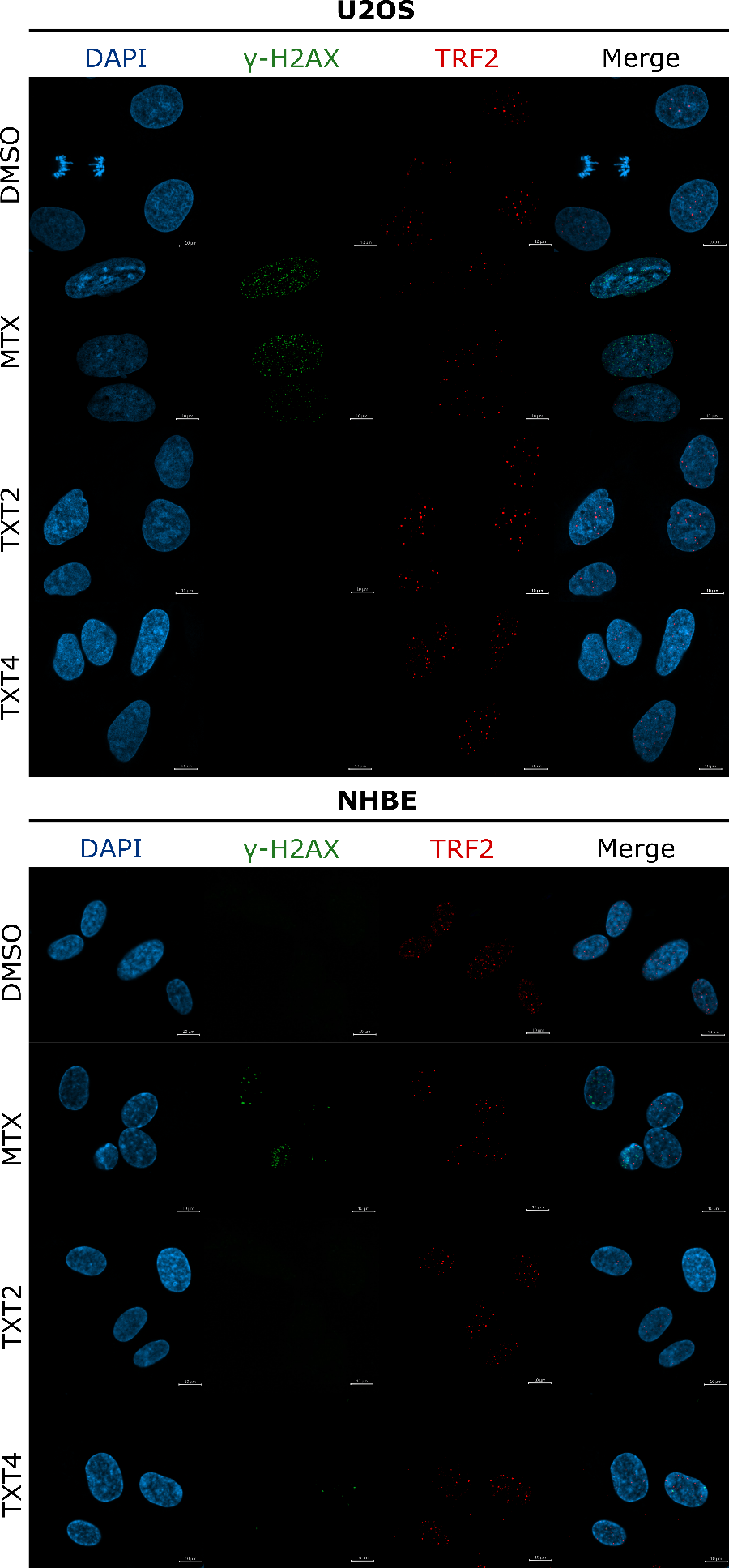
**

**Figure S6** Representative high-resolution laser scanning confocal images showing the γ-H2AX with TRF2 after treatment NHBE and U2OS cell lines. Scale bars =10 μm. Nuclei were counterstained with DAPI.

**
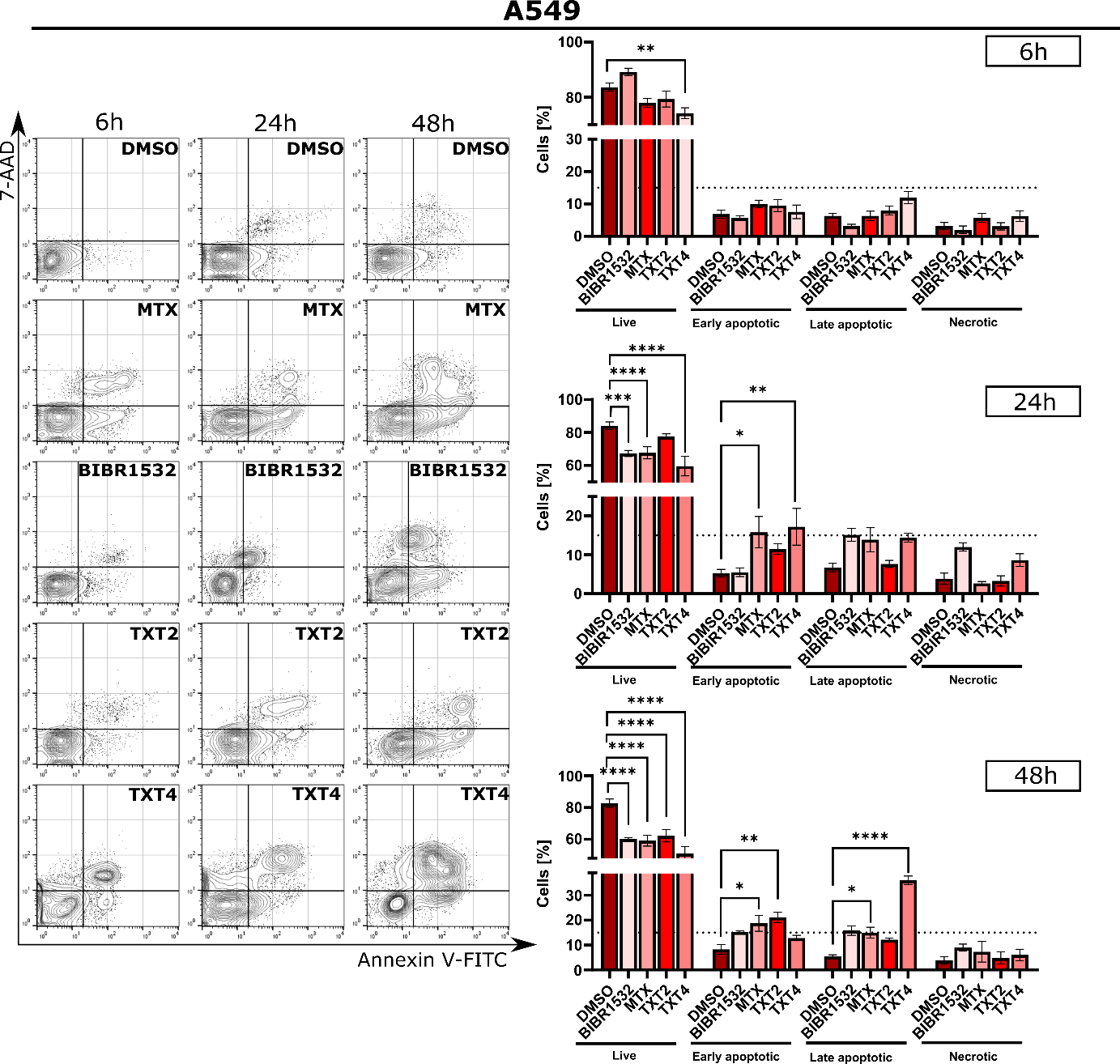
**

**Figure S7** Flow cytometric analysis of A549 cell line after 6, 24, and 48 h of treatment with TXT compounds, using Annexin V-FITC/7-AAD. DMSO was used as a negative control, whereas MTX and BIBR1532 were used as positive controls. Representative dot-plots are presented on the left panel and the quantitation of analysis is presented on a bar graph. Data represent mean values ± s.d. from at least three independent experiments. *p<0.01, **p<0.001, ***p<0.0001, and ****p<0.00001 (two-way ANOVA and post hoc Dunnett’s test).

**
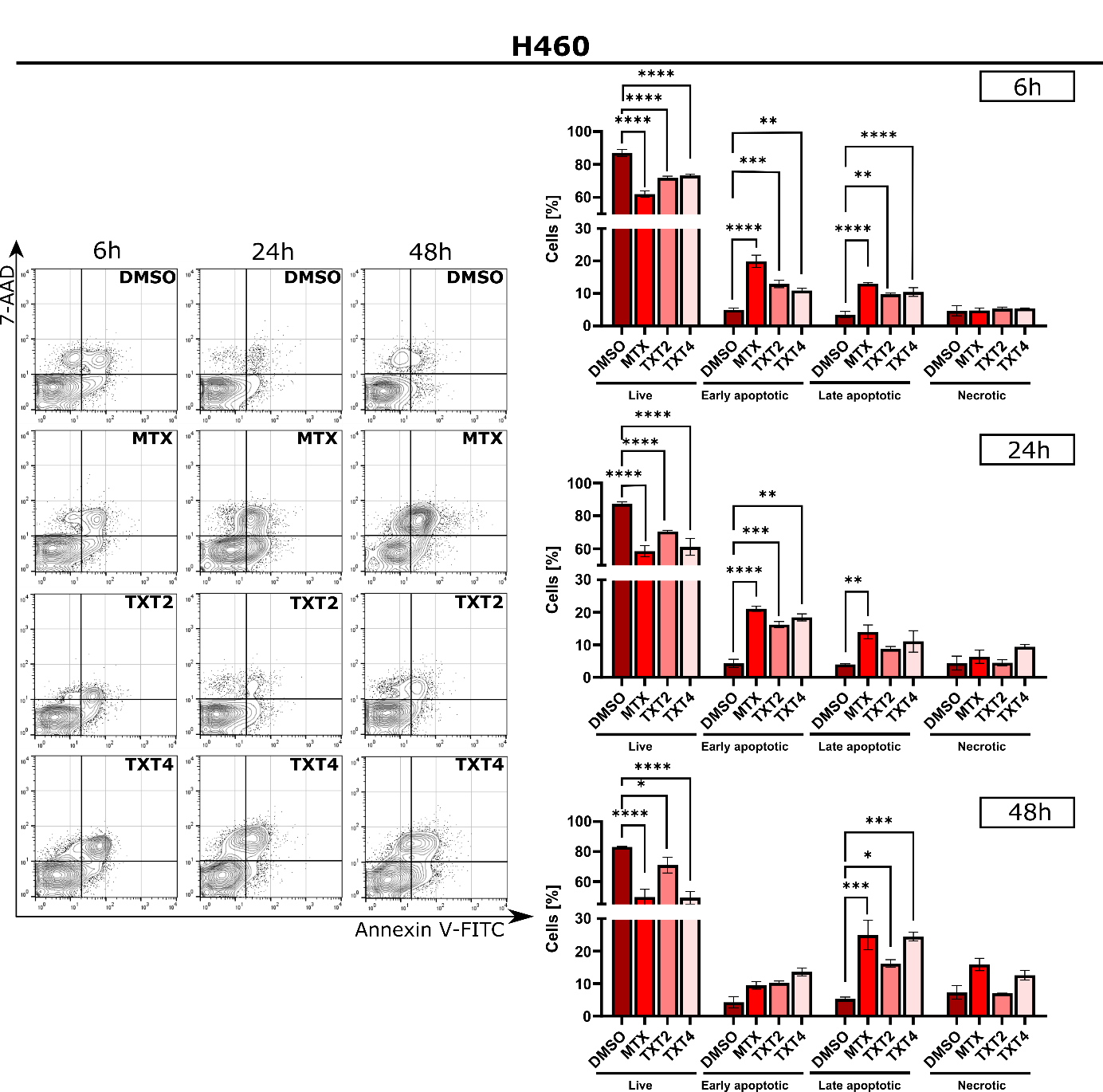
**

**Figure S8** Flow cytometric analysis of H460 cell line after 6, 24, and 48 h of treatment with TXT compounds, using Annexin V-FITC/7-AAD. DMSO and MTX were used as a negative and positive control, respectively. Representative dot-plots are presented on the left panel and the quantitation of analysis is presented on a bar graph. Data represent mean values ± s.d. from at least three independent experiments. *p<0.01, **p<0.001, ***p<0.0001, and ****p<0.00001 (two-way ANOVA and post hoc Dunnett’s test).

**
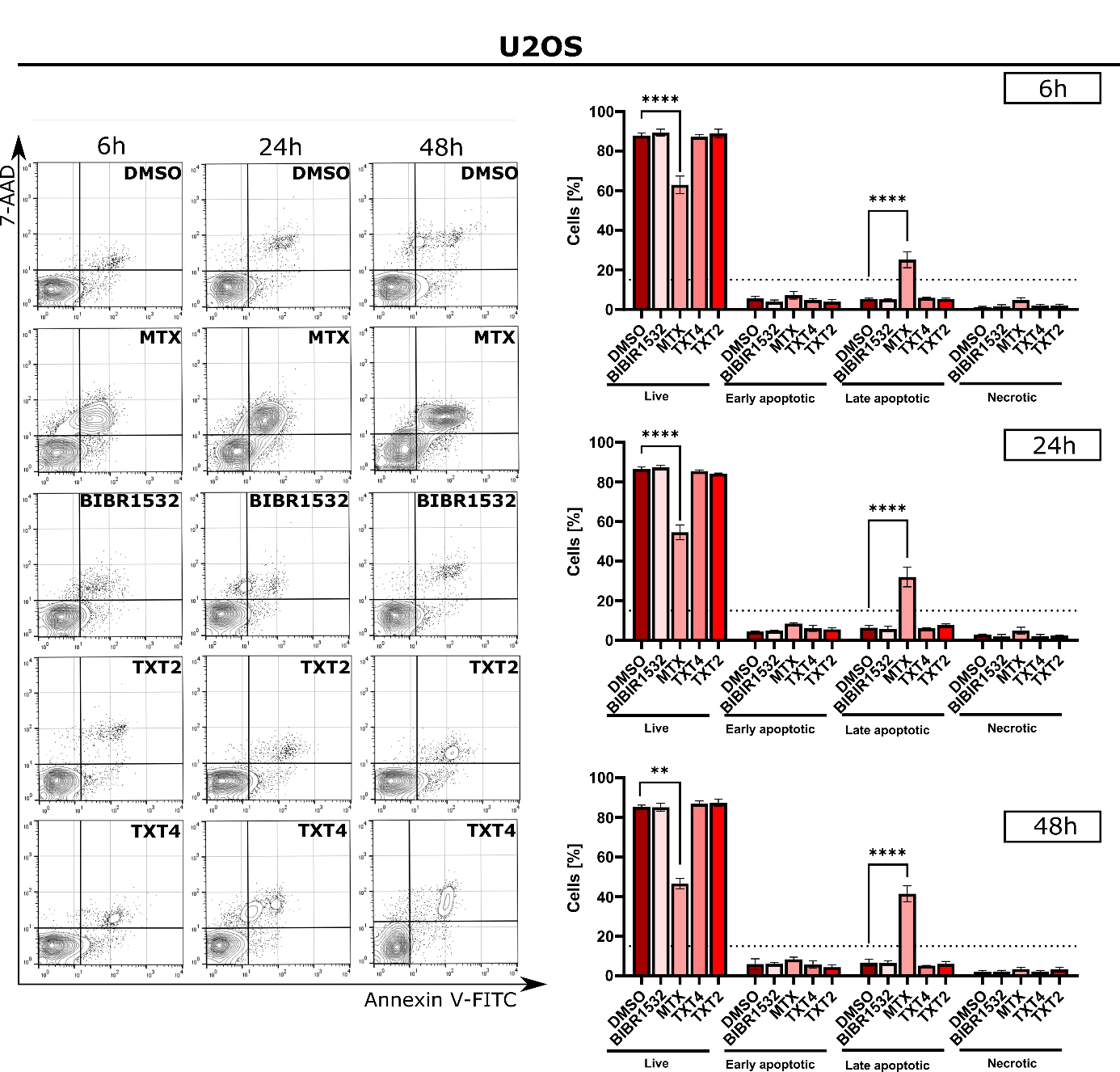
**

**Figure S9** Flow cytometric analysis of U2OS cell line after 6, 24, and 48 h of treatment with TXT compounds, using Annexin V-FITC/7-AAD. DMSO was used as a negative control, whereas MTX and BIBR1532 were used as positive controls. Representative dot-plots are presented on the left panel and the quantitation of analysis is presented on a bar graph. Data represent mean values ± s.d. from at least three independent experiments. **p<0.001, ***p<0.0001, and ****p<0.00001 (two-way ANOVA and post hoc Dunnett’s test).

**
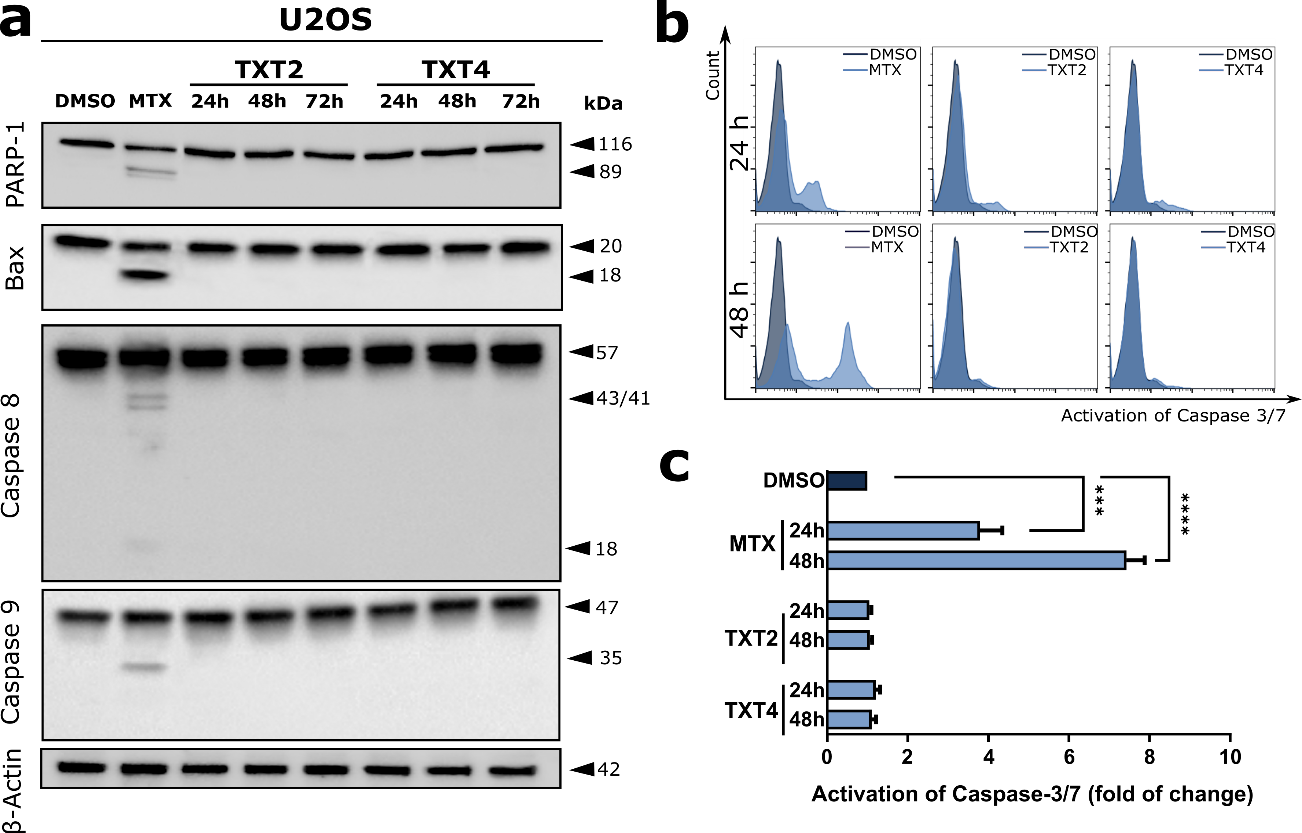
**

**Figure S10** The exposure of TERT-negative cells to TXT compounds did not induce apoptotic cell death. **a**. Representative western immunoblotting showing the amounts of apoptotic-related proteins in U2OS cell lines exposed to TXT compounds. DMSO and MTX were used as negative and positive controls, respectively. β-actin was used to ensure equal protein loading. Numbers on the right indicate the molecular weight (kDa); **b.** Representative histograms of flow cytometric analyses of caspase-3/7 activation in U2OS cell lines at 24 and 48 h of treatment with TXT compounds. DMSO and MTX were used as negative and positive controls, respectively; **c**. Quantitation of caspase-3/7 activation as assessed by flow cytometry. Data have been reported as fold-change of caspases activation with respect to DMSO-treated cells and represent mean values ±  s.d. from at least three independent experiments. ***p<0.0001, and ****p<0.00001 (one-way ANOVA and post hoc Dunnett’s test).

**
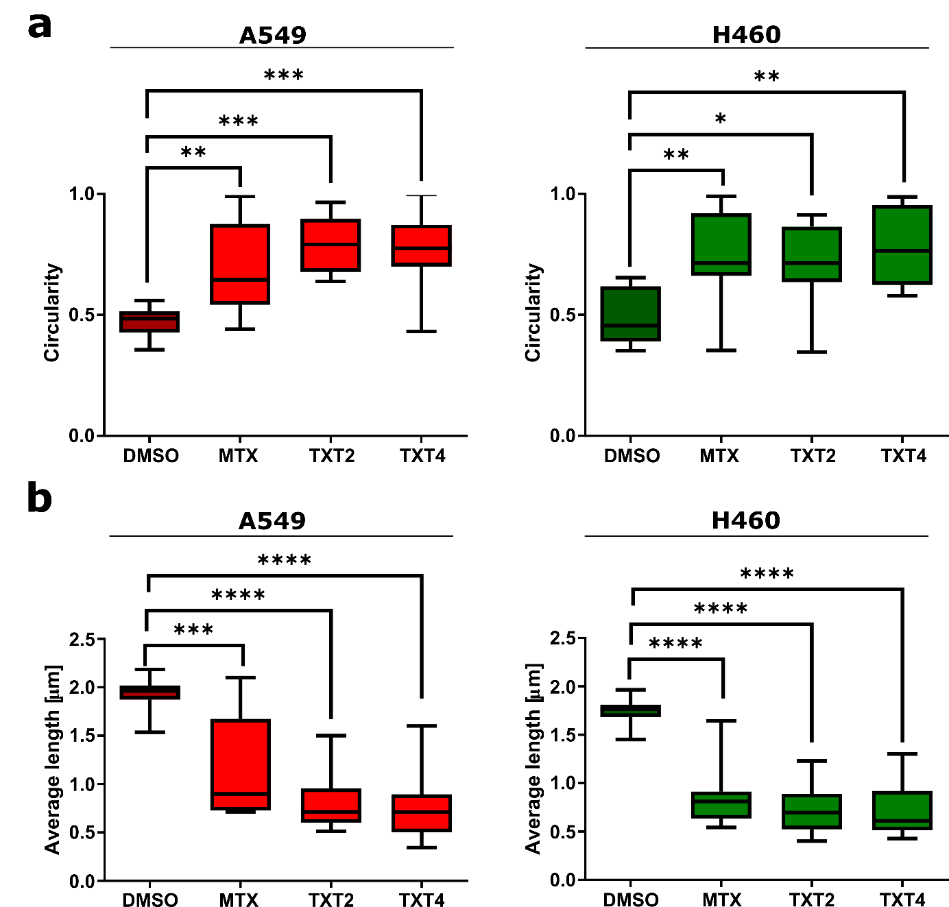
**

**Figure S11** Quantitative mitochondrial morphometric analyses of images which are presented in Figure 5d of the main article. **a**. Circularity of mitochondria (index of elongation); **b.** Average length (μm) of mitochondria. Error bars represent the s.d*.* of data obtained in at least n=15 randomly selected locations on the slide. *p<0.01, **p<0.001, ***p<0.0001, and ****p<0.00001 compared to DMSO (one-way ANOVA and post hoc Dunnett’s test).

**
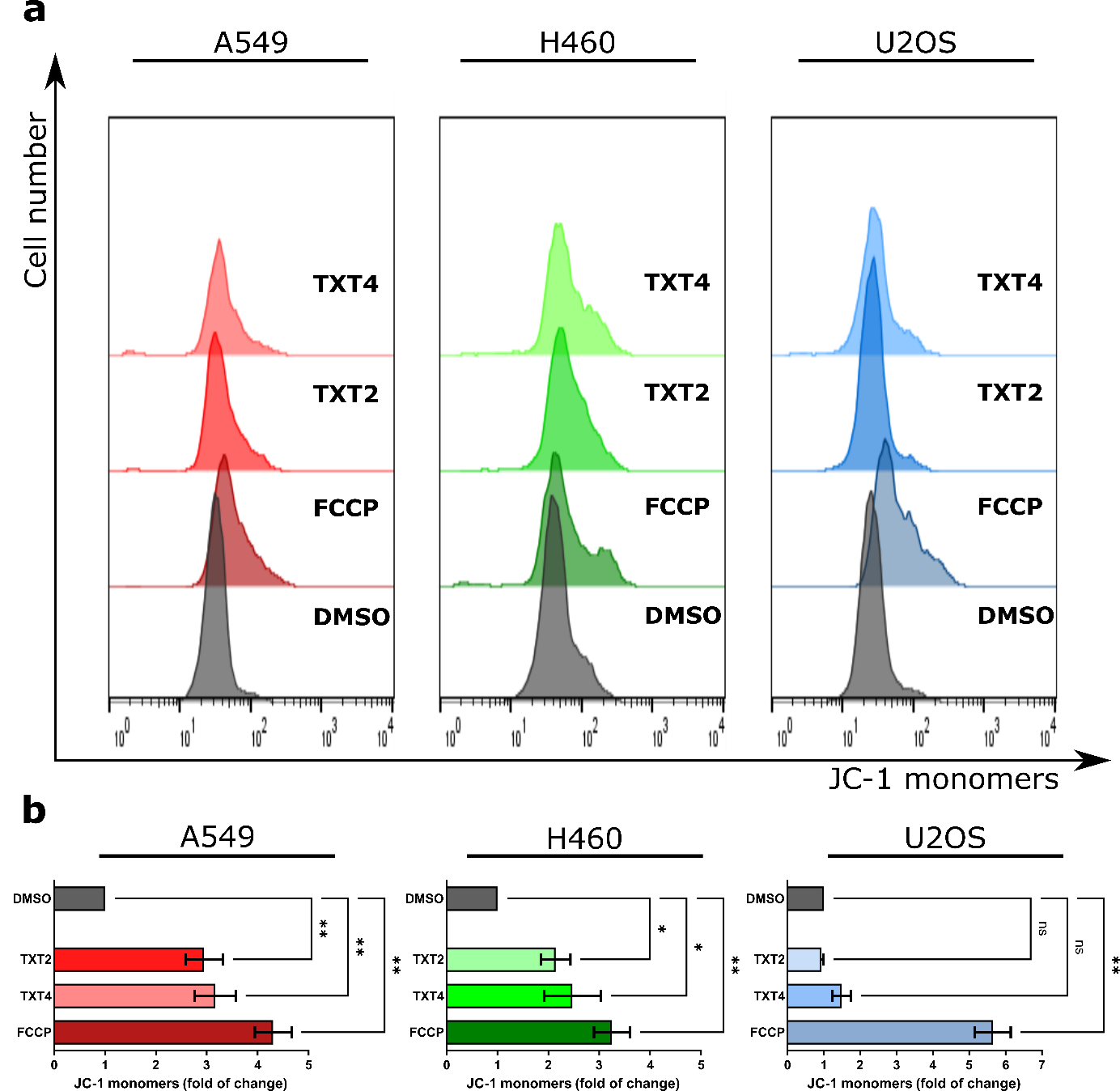
**

**Figure S12** Analysis of mitochondrial membrane potential after 24 h of treatment NSCLC and U2OS cells with TXT compounds. DMSO and FCCP were used as negative and positive control, respectively. **a.** Representative histograms; **b.** Quantitation of analyses as assessed by flow cytometry. Data have been reported as fold-change of JC-1 monomers with respect to DMSO-treated cells and represent mean values ±  s.d. from at least three independent experiments. ***p<0.0001, and ****p<0.00001 (one-way ANOVA and post hoc Dunnett’s test).
