# Supplementary material for: Teloxantron inhibits the processivity of telomerase with preferential DNA damage on telomeres
Source: Cell Death Dis. 2022 Nov 28;13(11):1005. doi: 10.1038/s41419-022-05443-y (PMC9701690; doi:10.1038/s41419-022-05443-y)
Supplement: Supplementary file 3 — Supplemental materials and methods [file 41419_2022_5443_MOESM3_ESM.docx]

**Supplementary material and methods**

*Cell cultivation*

NSCLC A-549 and H460 cells, human osteosarcoma U2OS cells, and normal human bronchial epithelial cells NHBE2594 were obtained from the American Type Culture Collection. All cell lines were maintained in different media supplemented with 10% fetal bovine serum (Corning, New York, NY, USA), 2 mM L-glutamine (Corning), and antibiotics (penicillin 62.6 µg/ml and streptomycin 40 µg/ml; Sigma-Aldrich) at 37°C in a humidified atmosphere containing 5% CO_2_ and were routinely screened for Mycoplasma contamination. NSCLC cells were cultured in RPMI-1640 medium (Corning), U2OS cells in McCoy’s 5A medium (Corning), and NHBE2594 cells in Bronchial Epithelial Cell Growth Medium (Lonza, Walkersville, MD, USA). The culture media used for maintaining normal cell lines contained supplements and growth factors, as per the manufacturer’s instructions.

*TRAP*

Cells (1.6×10^5^) were cultured overnight to allow attachment. On the next day, cells were collected and lysed with NP-40 buffer (10 mM Tris base (pH 8), 1 mM ethylene glycol-bis(β-aminoethyl ether)-N,N,N′,N′-tetraacetic acid, 1 mM ethylenediaminetetraacetic acid, 1% (v/v) Nonidet-P40, 0.25 mM sodium deoxycholate, 10% (v/v) glycerol, 150 mM NaCl, 5 mM 2-mercaptoethanol, 0.1 M 4-(2-aminoethyl) benzenesulfonyl fluoride hydrochloride). Protein concentration in cells was determined using the DC Protein Assay Kit (Bio-Rad, Hercules, CA, USA), and the samples were stored at –80°C until analysis. PCR was performed in TRAP buffer (200 mM Tris base (pH 8.3), 15 mM MgCl_2_, 630 mM KCl, 0.5% (v/v) Tween 20 (Thermo Fisher Scientific, Waltham, MA, USA)) with 0.1 mg/ml BSA, 50 µM dNTPs, oligonucleotides (Cy5-TS (5′-AATCCGTCGAGCAGA GTT-3′), TSNT (5′-AATCCGTCGAGCAGAGTTAAAAGGCCGAGAAGCGAT-3′), NT (5′-ATCGCTTCTCGGCCT TTT-3′), ACX (5′-GCGCGGCTTACCCTTACCCTTACCCTAACC-3′)), 2 U Taq polymerase (Thermo Fisher Scientific), 0.1 µg of protein, and 0.4% DMSO or compounds. After telomerase elongation and denaturation for 40 min at 25°C and 5 min at 95°C, respectively, PCR was conducted in 30 cycles (95°C, 30 s; 52°C, 30 s; and 72°C, 45 s). The PCR products were separated by electrophoresis on 8% nondenaturing polyacrylamide gel at 110 V for 4.5 h. Then, the gel was fixed in 0.5 M NaCl, 50% ethanol, and 40 mM sodium acetate (pH 4.2) for 15 min at RT, and subsequently visualized using ChemiDoc XRS+ Imaging System (Bio-Rad). The band intensities of PCR products were measured using Image Lab Software 6.0.1 (Bio-Rad) and quantitatively analyzed by densitometry. Relative telomerase activity (RTA) was calculated using the following formula: RTA=(XTP/XIT)/(CTP/CIT)×100%, where X and C are the band intensities of samples and telomerase positive control, respectively. All oligonucleotides were purchased from Genomed (Warsaw, Poland), and other reagents and materials from Sigma-Aldrich unless otherwise stated.

*FRET*

The initial DNA melting screen was performed using dual-labeled G4-forming sequence from the human telomere (5′-FAM-[GGG-TTA-GGG-TTA-GGG-TTA-GGG]-TAMRA-3′) (Genomed), which forms a well-characterized parallel stranded G4 structure. Oligonucleotide was prepared as a 400 nM solution in 10 mM sodium cacodylate buffer (pH 7.2) with 90 mM NaCl and 10 mM KCl, and the solution was thermally annealed by heating at 95°C for 5 min and allowed to cool overnight to RT. On the next day, equal volumes of the probe were added to the compound solutions in DMSO, to achieve the following relative compound:probe ratios: 10:1, 5:1, 2:1, 1:1, and 0.5:1. The solutions were distributed in 96-well plates (Roche Life Science, Penzberg, Germany) to obtain a total reaction volume of 20 μl. A probe solution containing 0.5% DMSO was used as a negative control. Measurements were carried out in triplicate using a LightCycler 480 thermocycler (Roche Life Science) with a filter setting of 465 nm/510 nm at a range of 30–95°C with a rate of 0.01°C/s. Melting points were determined using GraphPad version 9.0 software (San Diego, CA, USA) and the Hill model with a variable coefficient.

*Molecular docking analyses*

The protein structure PDB 3DU6 of *Tribolium castaneum* TERT which shares a high degree of homology with hTERT was used for docking analyses.^1^ Docking simulations and estimation of ligand-receptor affinity were performed using the Autodock Vina program.^2^ The maximum energy difference between the worst and best docking modes was set to 5 kcal/mol. Two grids were selected for Vina docking, of which one was the center of mass of protein 3DU6 and the other was the TERT active site consisting of three invariant aspartic acid residues (D251, D343, and D344)(1). The grid size in docking was set to 40 Å×68 Å×74 Å for all proteins and 30 Å×26 Å×20 Å for the enzyme active site. Additionally, the rigid receptor and flexible ligands were parametrized using AutodockTools 1.5.6, and the parameterized systems were recorded in the PDBQT file.^3^ In particular, both receptor and ligand were presented using the united atom model involving nonpolar hydrogen atoms. Atomic charges were estimated using the Gasteiger−Marsili method.^4^ Results figures were created by the VMD software.^5^

*Induction of DNA DSBs*

Cells (1.5×10^6^) were seeded onto tissue culture plates and allowed to attach overnight. On the next day, cells were exposed to the tested compounds for the indicated time. MTX and BIBR1532 were used as reference compounds. After treatment, cells were collected by trypsinization, fixed in 75% ethanol, and stored at −20°C until analysis. Then, cells were rehydrated with PBS on ice for 5 min, permeabilized in 0.2% Triton X-100 in PBS for 15 min at RT, and labeled with Alexa Fluor 488-conjugated mouse anti-p-γH2AX (Ser139) antibody (#613406, 1:200 dilution; BioLegend, San Diego, CA, USA) for 1.5 h at 37°C. Then, cells were washed with PBS and stained with 20 μg/μl propidium iodide and 50 μg/μl RNase (Thermo Fisher Scientific) for 20 min. Analysis was performed using a Guava EasyCyte 8 cell sorter (Merck Millipore, Burlington, MA, USA) and FlowJo v10 software (BD Life Sciences, Ashland, OR, USA).

*Apoptosis and caspase 3/7 assay*

Cells were seeded onto tissue culture plates and allowed to attach overnight. Day after cells were treated with compounds at IC_90_ concentrations for the indicated time. After incubation with the compounds, cells were harvested by trypsinization, rinsed twice with PBS, and stained with the 7-AAD (#A1310, Thermo Fisher Scientific) and Annexin V FITC conjugate (#A13199, Thermo Fisher Scientific) for apoptosis assay and with CellEvent™ Caspase-3/7 Green Flow Cytometry Assay Kit (#C10427, Thermo Fisher Scientific) reagent in the CellEvent™ Caspase-3/7 Green Flow Cytometry Assay Kit (#C10427; Thermo Fisher Scientific) for caspase-3/7 activation according to the manufacturer’s protocols. Analysis was performed with a Guava EasyCyte 8 cell sorter (Merck Millipore) and FlowJo v10 software. Each experiment was repeated at least three times.

*Mitochondrial membrane potential*

Cells were seeded onto tissue culture plates and allowed to attach overnight. Day after, cells were treated with compounds at IC_90_ concentrations for 24 h. The positive control consisted of 10 µM carbonyl cyanide-p-trifluoromethoxyphenylhydrazone (FCCP; Sigma-Aldrich). After treatment, the culture medium was replaced by a fresh medium supplemented with 5 µg/ml JC‑1 dye (Sigma-Aldrich), and the cells were incubated in dark for 20 min at 37°C. Subsequently, the cells were harvested by trypsinization, washed twice with PBS, and measured using a Guava EasyCyte 8 cell sorter (Merck Millipore) and FlowJo v10 software. Each experiment was repeated at least three times.

*Live cell imaging*

For the analysis of mitochondrial morphology, cells were grown on a covered glass-bottomed 12-well plate and allowed to attach overnight. On the next day, cells were exposed for 6 h to the tested compounds at their IC_90_ concentration or to DMSO. Then, mitochondrial labeling was performed by incubating the cells with MitoTracker Green FM probe (Thermo Fisher Scientific) and Hoechst 33342 (Thermo Fisher Scientific) for 15 min, according to the manufacturer’s instructions. After staining, the cells were suspended in a fresh growth medium. Images were acquired with an LSM 800 inverted laser scanning confocal microscope (Carl Zeiss, Jena, German), equipped with an Airyscan detector for high-resolution confocal scanning using a ×63 1.4 NA Plan Apochromat objective (Carl Zeiss). Cells were incubated in an incubation chamber at 37°C with 5% CO_2_. During imaging, the parameters laser intensity, exposure times, gain settings, and so on were kept constant for both compound-treated and DMSO-treated cells.

*Mitochondrial morphometric analyses*

Analysis of mitochondrial circularity was performed using the mitochondrial morphology plugin of ImageJ (NIH) developed by Dagda et al.^6^ The mitochondrial length was analyzed with a macro developed by Merrill et al.^7^ Data were obtained in at least n=15 randomly selected locations on the slide.

*Western blot*

The protein extract (30 µg) was separated by 10% sodium dodecyl sulfate-polyacrylamide gel electrophoresis, transferred onto a microporous polyvinylidene difluoride membrane (Bio-Rad), and incubated with primary antibodies. The results of the analysis of protein expression using antibodies are presented in **Table S1**. Blots were incubated with an appropriate peroxidase-conjugated secondary antibody, and proteins were detected using an enhanced chemiluminescence detection reagent kit (Thermo Fisher Scientific) and a ChemiDoc XRS+ Imaging System (Bio-Rad). Band intensity was measured using Image Lab Software 5.2 (Bio-Rad).

**Table S1** List of antibodies used in Western Blot

| Antibody name | Company | Dilution |
| --- | --- | --- |
| Anti-Phospho-ATR (Ser428) (#2853) | Cell Signaling | 1:1000 |
| Anti-Phospho-Chk2 (Thr68) (C13C1) (#2197) | Cell Signaling | 1:1000 |
| Anti-Phospho-Chk1 (Ser345) (133D3) (#2348) | Cell Signaling | 1:1000 |
| Anti-Phospho-Histone H2A.X (Ser139) (20E3) (#9718) | Cell Signaling | 1:1000 |
| Anti-Phospho-ATM (Ser1981) (D6H9) (#5883) | Cell Signaling | 1:1000 |
| Anti-ATR (ab10312) | Abcam | 1:1000 |
| Anti-ATM (2C1 [1A1]) (ab78) | Abcam | 1:1000 |
| Anti-Caspase-3 (#9662) | Cell Signaling | 1:1000 |
| Anti-Caspase-9 (#9502) | Cell Signaling | 1:1000 |
| Anti-Caspase-8 (1C12) (#9746) | Cell Signaling | 1:1000 |
| Anti-PARP (#9542) | Cell Signaling | 1:1000 |
| Anti-Bcl-2 (#4223) | Cell Signaling | 1:1000 |
| Anti-BID (#2002) | Cell Signaling | 1:1000 |
| Anti-Bax (#2772) | Cell Signaling | 1:1000 |
| Anti-TERT (Y182) (ab32020) | Abcam | 1:1000 |
| Anti-actin (sc-1616) | Santa Cruz | 1:100 |
| Anti-mouse-HRP (715-035-150) | Jacson ImmunoResearch Labs | 1:10000 |
| Anti-rabbit-HRP (711-035-152) | Jacson ImmunoResearch Labs | 1:10000 |
| Anti-goat-HRP (705-036-147) | Jacson ImmunoResearch Labs | 1:10000 |

*Statistical analyses*

Statistical analyses were performed using GraphPad Prism 9 software (Graph Pad Software, San Diego, CA, USA). The sample sizes required for the experiments were estimated based on the preliminary results. Data were obtained from at least three independent experiments and are presented as mean ± s.d. One-way ANOVA or two-way ANOVA were performed for multiple comparisons, corrected using the Dunnett’s test. P < 0.05 was considered significant.

**References**

1. Gillis AJ, Schuller AP, Skordalakes E. Structure of the Tribolium castaneum telomerase catalytic subunit TERT. Nature. 2008;455(7213):633-637.

2. Wang R, Lai L, Wang S. Further development and validation of empirical scoring functions for structure-based binding affinity prediction. J Comput Aided Mol Des. 2002;16(1):11-26.

3. Morris GM, Huey R, Lindstrom W, Sanner MF, Belew RK, Goodsell DS, et al. AutoDock4 and AutoDockTools4: Automated docking with selective receptor flexibility. J Comput Chem. 2009;30(16):2785-2791.

4. Gasteiger J, Marsili M. A new model for calculating atomic charges in molecules. Tetrahedron Lett. 1978;19(34):3181–4.

5. Humphrey W, Dalke A, Schulten K. VMD: Visual molecular dynamics. J Mol Graph. 1996;14(1):33–8.

6. Dagda RK, Cherra SJ 3rd, Kulich SM, Tandon A, Park D, Chu CT. Loss of PINK1 function promotes mitophagy through effects on oxidative stress and mitochondrial fission. J Biol Chem. 2009;284(20):13843-13855.

7. Mortiboys H, Thomas KJ, Koopman WJ, Klaffke S, Abou-Sleiman P, Olpin S, et al. Mitochondrial function and morphology are impaired in parkin-mutant fibroblasts. Ann Neurol. 2008;64(5):555-565.
