# Supplementary material for: Teloxantron inhibits the processivity of telomerase with preferential DNA damage on telomeres
Source: Cell Death Dis. 2022 Nov 28;13(11):1005. doi: 10.1038/s41419-022-05443-y (PMC9701690; doi:10.1038/s41419-022-05443-y)
Supplement: Supplementary file 4 — Full-lenght gels and blots [file 41419_2022_5443_MOESM4_ESM.docx]

**
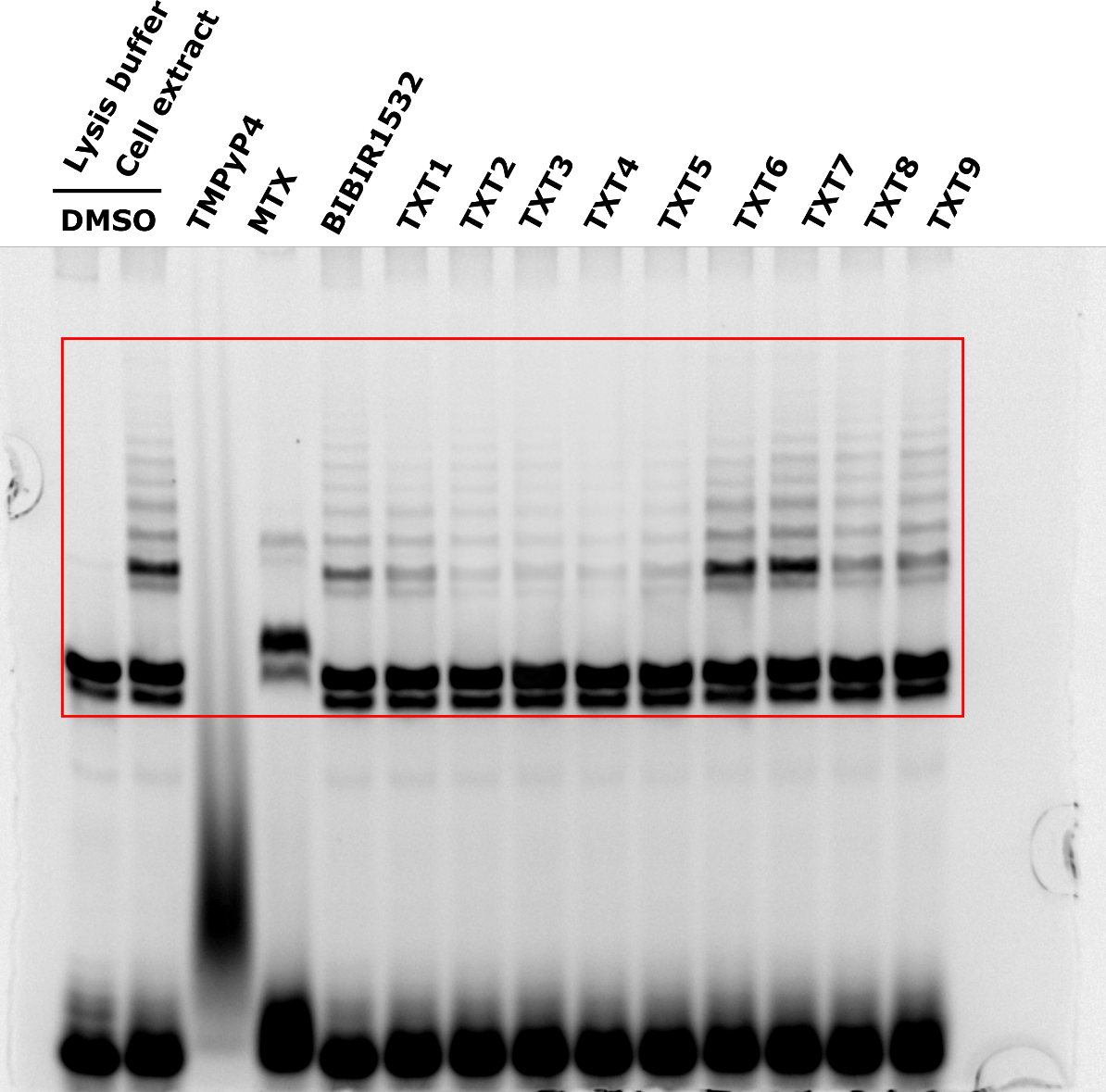
**

**Figure S13** Full-length gel presented in Figure 1 of the main article. The red box denotes the cropped regions of the gel.


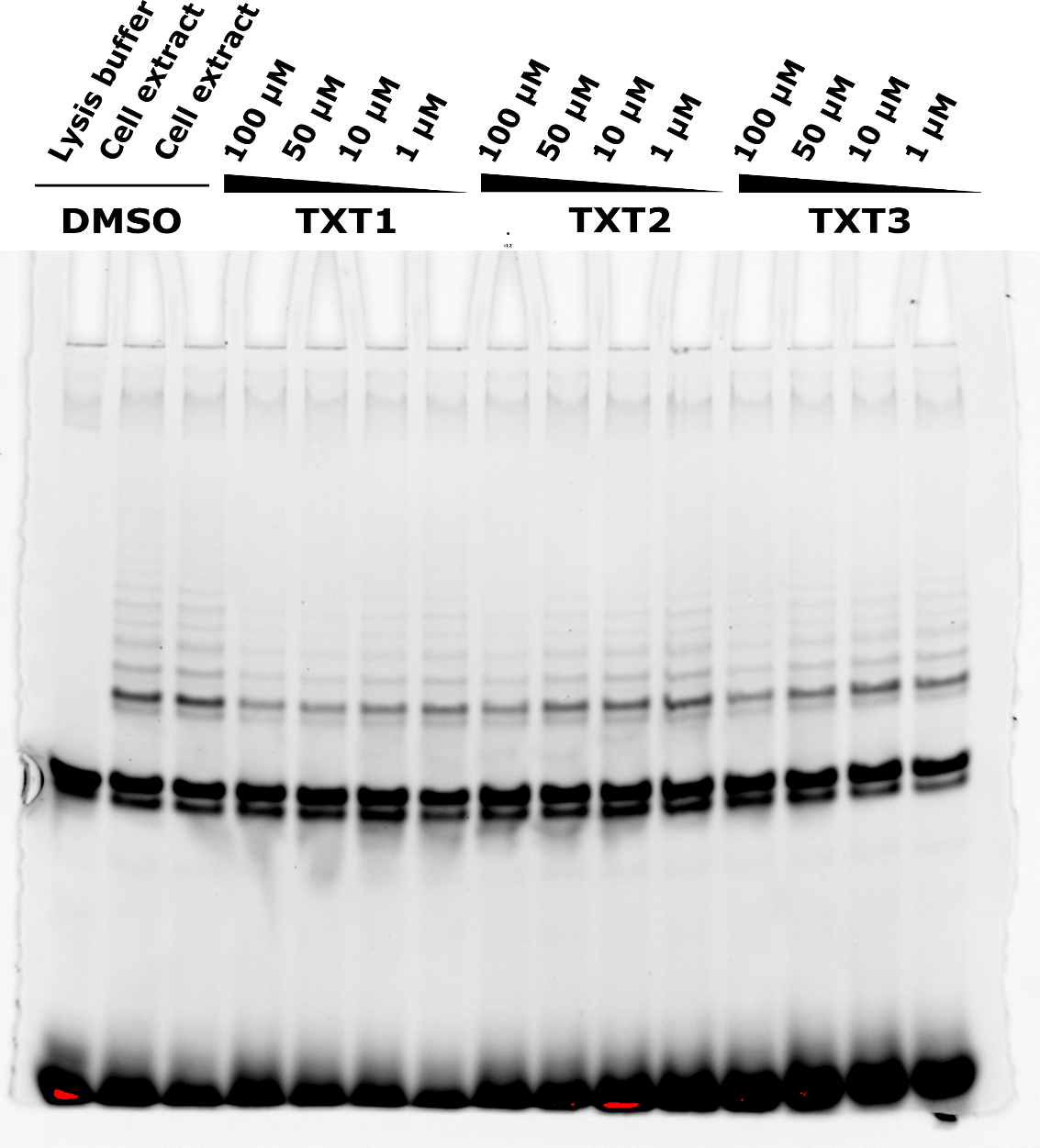


***Figure S14*** Full-length gel presented in Figure S1 of the Supplementary Information. The red box denotes the cropped regions of the gel.


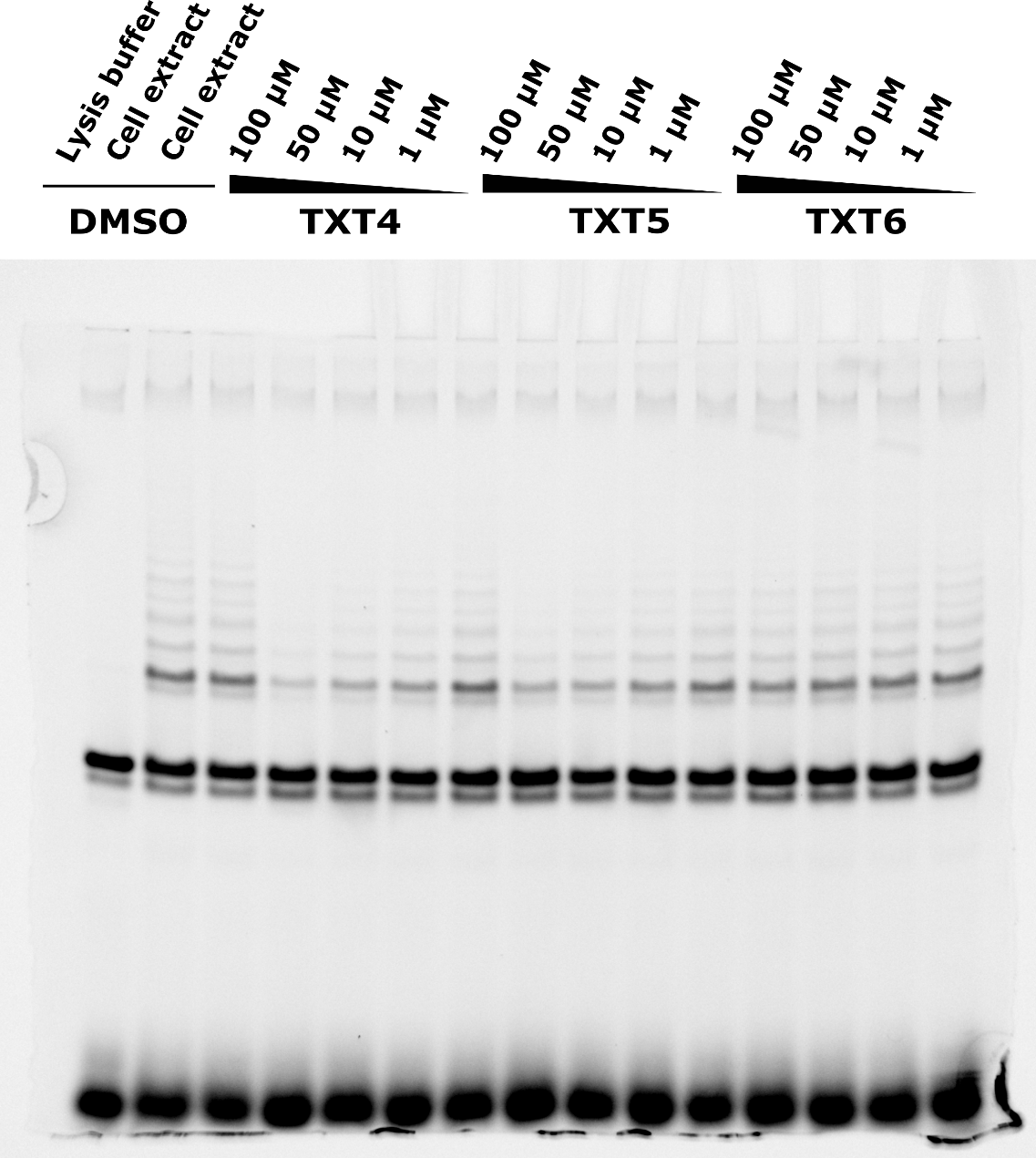


**Figure S15** Full-length gel presented in Figure S1 of the Supplementary Information. The red box denotes the cropped regions of the gel.


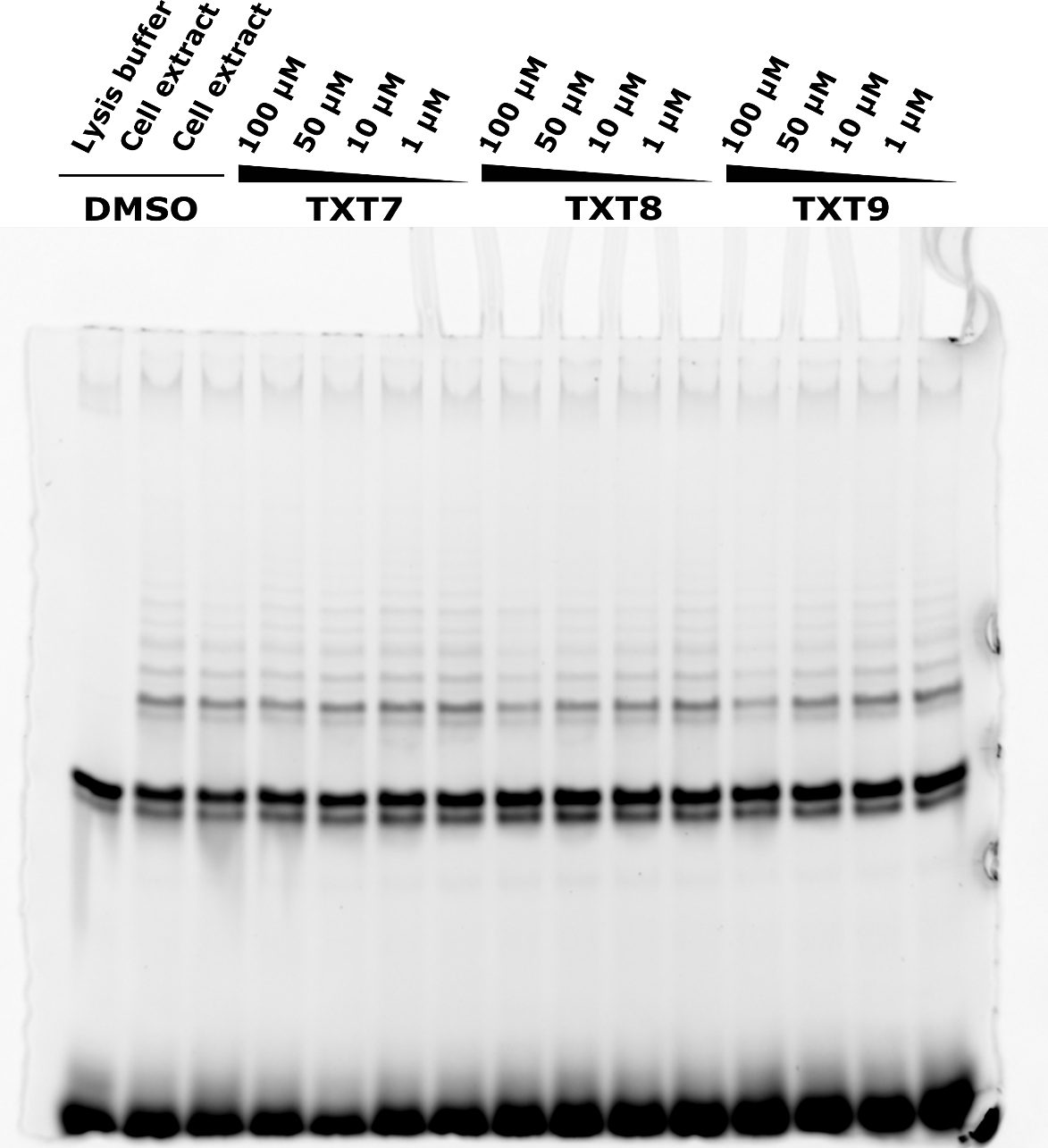


**Figure S16** Full-length gel presented in Figure S1 of the Supplementary Information. The red box denotes the cropped regions of the gel.


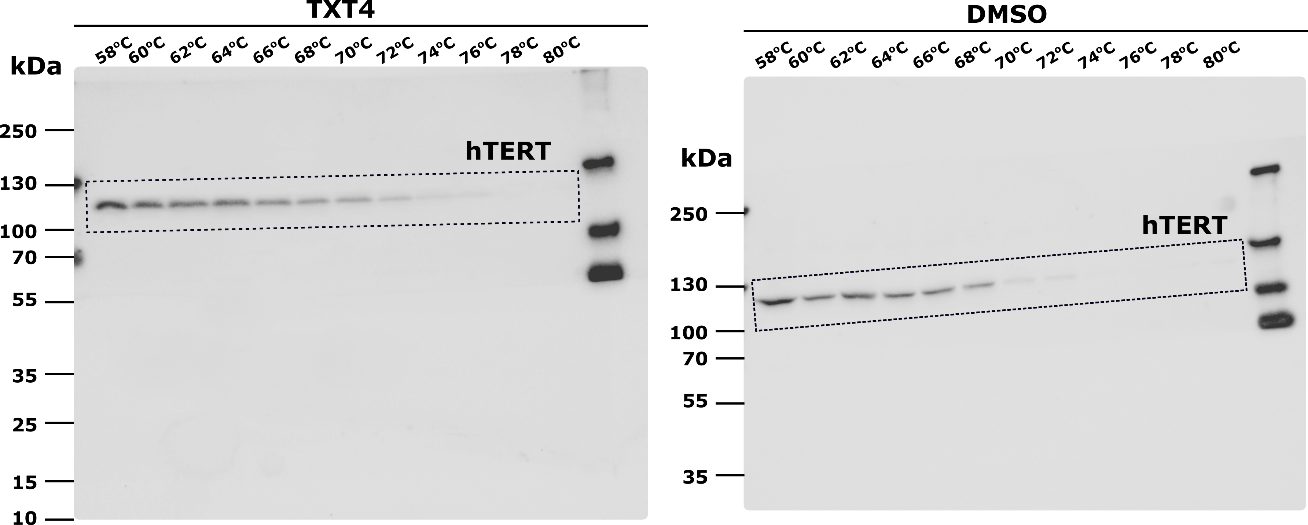


***Figure S17*** Full-length *blot* presented in Figure 2 of the main article. The dotted box denotes the cropped regions of the gel.


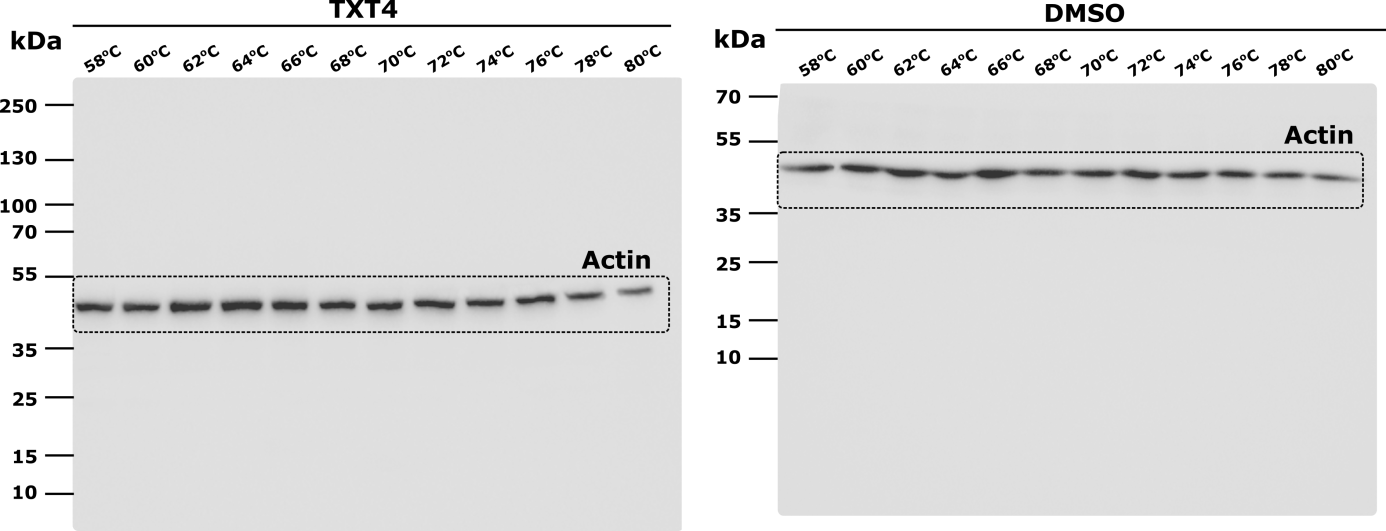


***Figure S18*** Full-length *blot* presented in Figure 2 of the main article. The dotted box denotes the cropped regions of the gel.


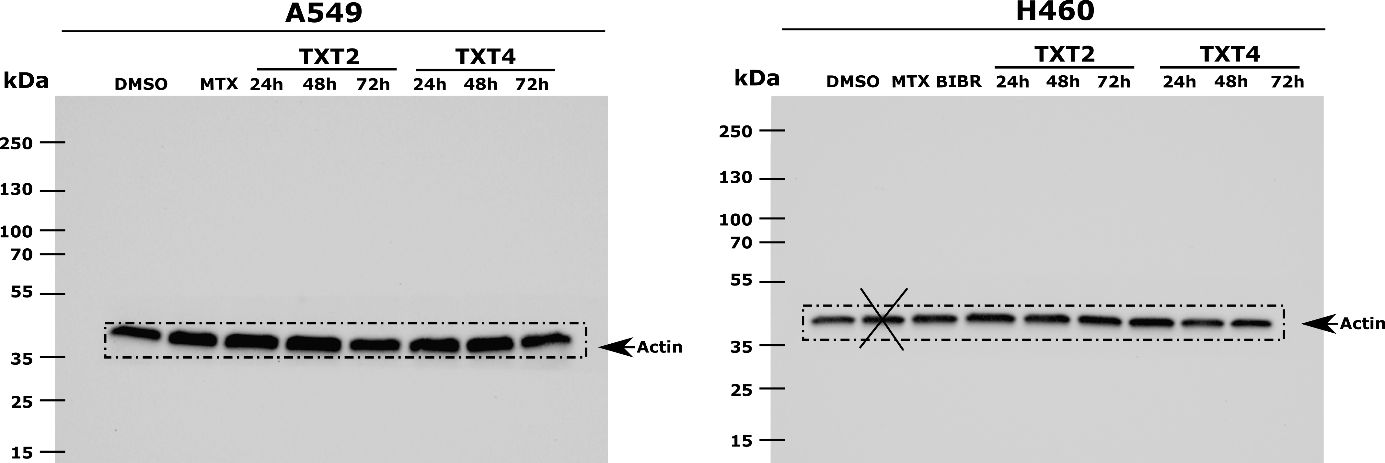


**Figure S19** Full-length blot presented in Figure 3 of the main article. The dotted box denotes the cropped regions of the blot.


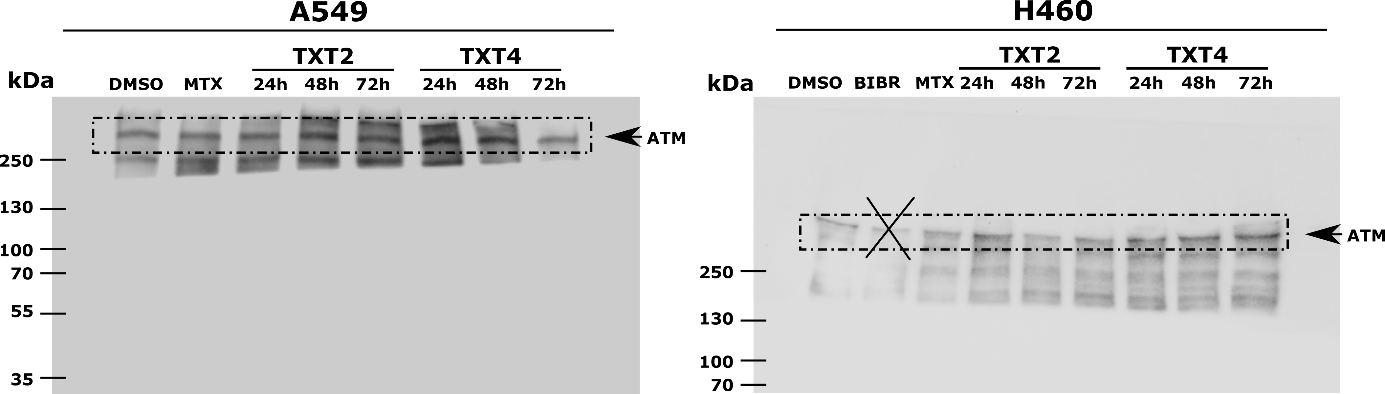


**Figure S20** Full-length blot presented in Figure 3 of the main article. The dotted box denotes the cropped regions of the blot.


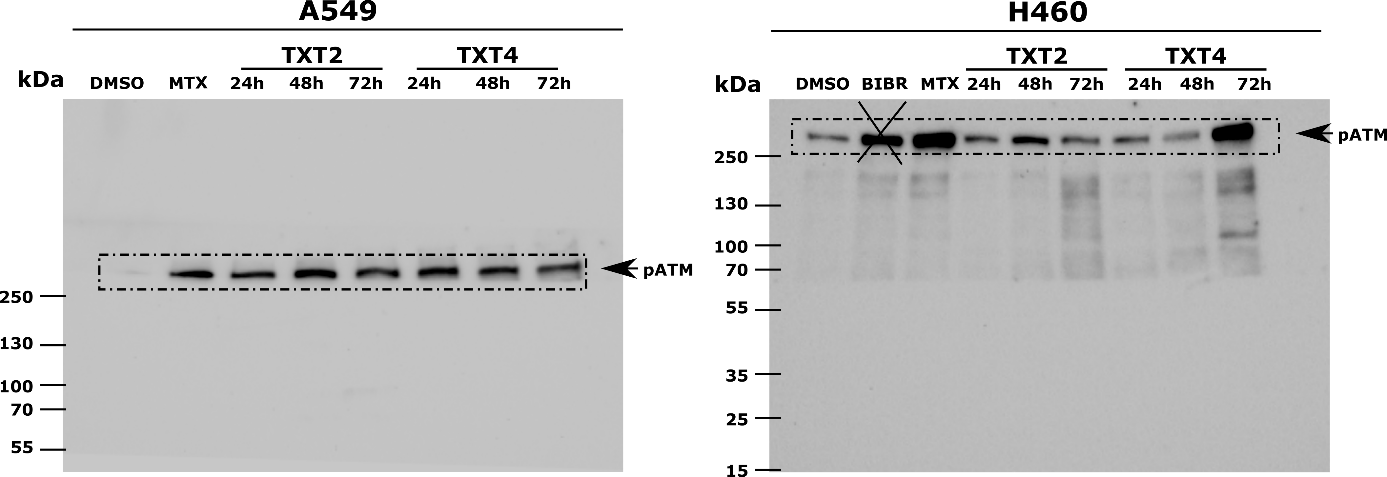


**Figure S21** Full-length blot presented in Figure 3 of the main article. The dotted box denotes the cropped regions of the blot.


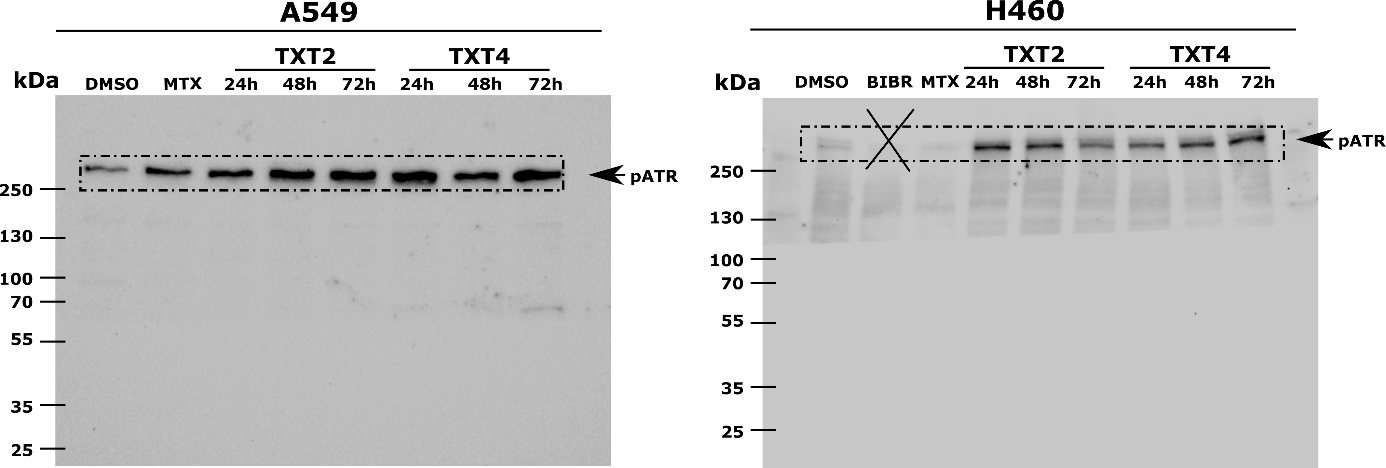


**Figure S22** Full-length blot presented in Figure 3 of the main article. The dotted box denotes the cropped regions of the blot.


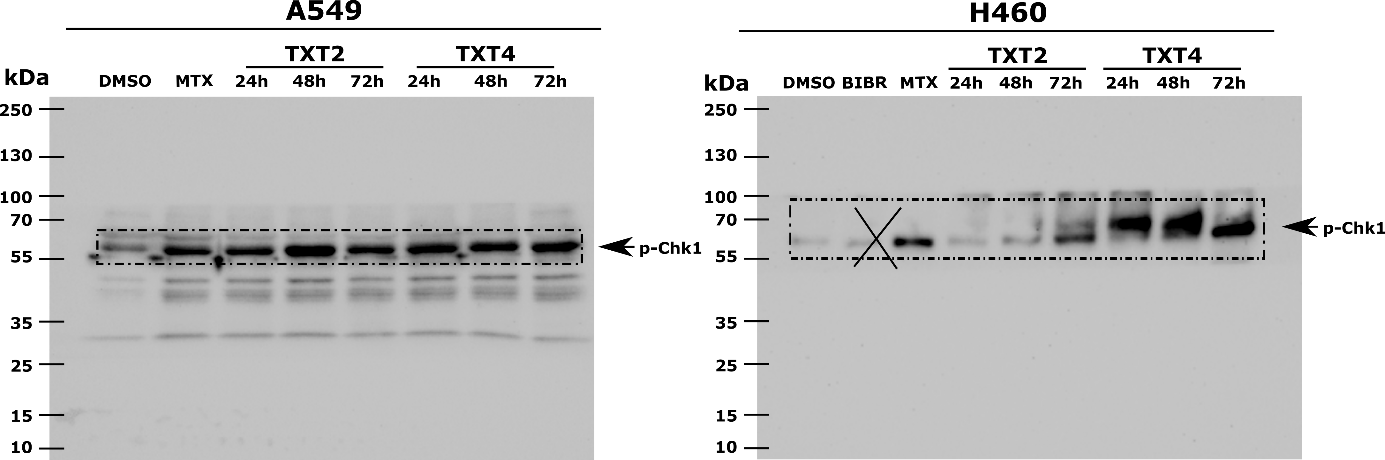


**Figure S23** Full-length blot presented in Figure 3 of the main article. The dotted box denotes the cropped regions of the blot.


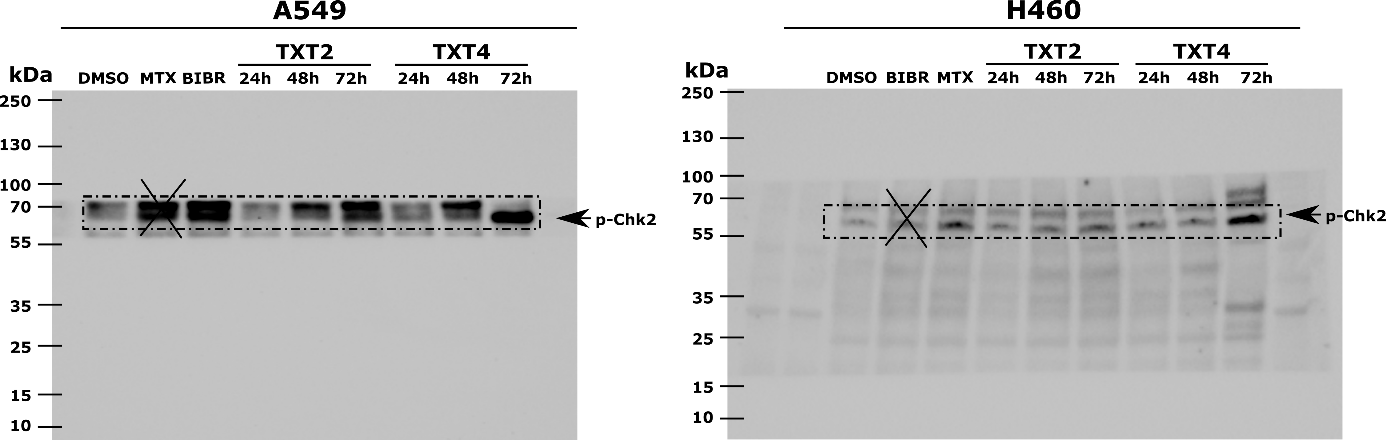


**Figure S24** Full-length blot presented in Figure 3 of the main article. The dotted box denotes the cropped regions of the blot.


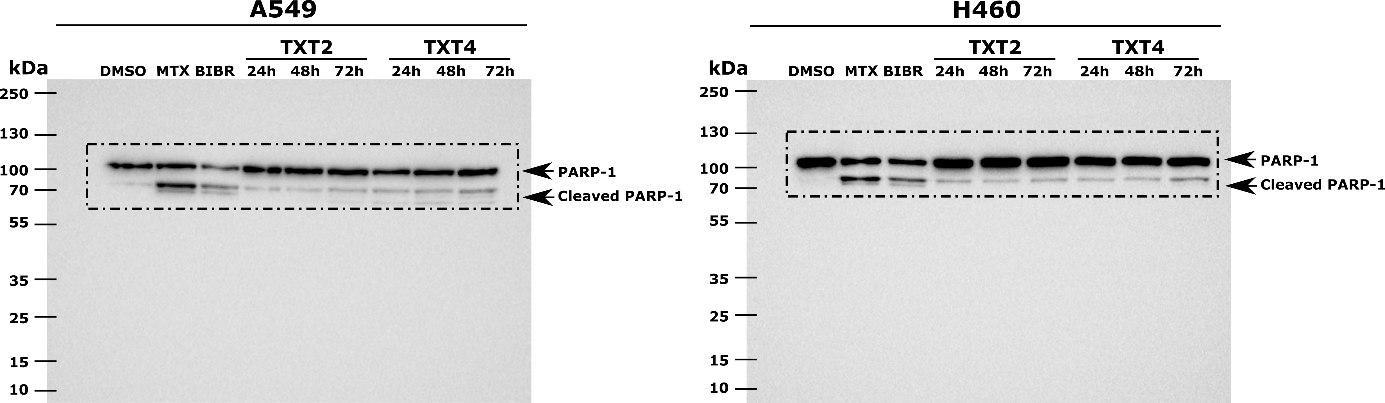


**Figure S25** Full-length blot presented in Figure 5 of the main article. The dotted box denotes the cropped regions of the blot.


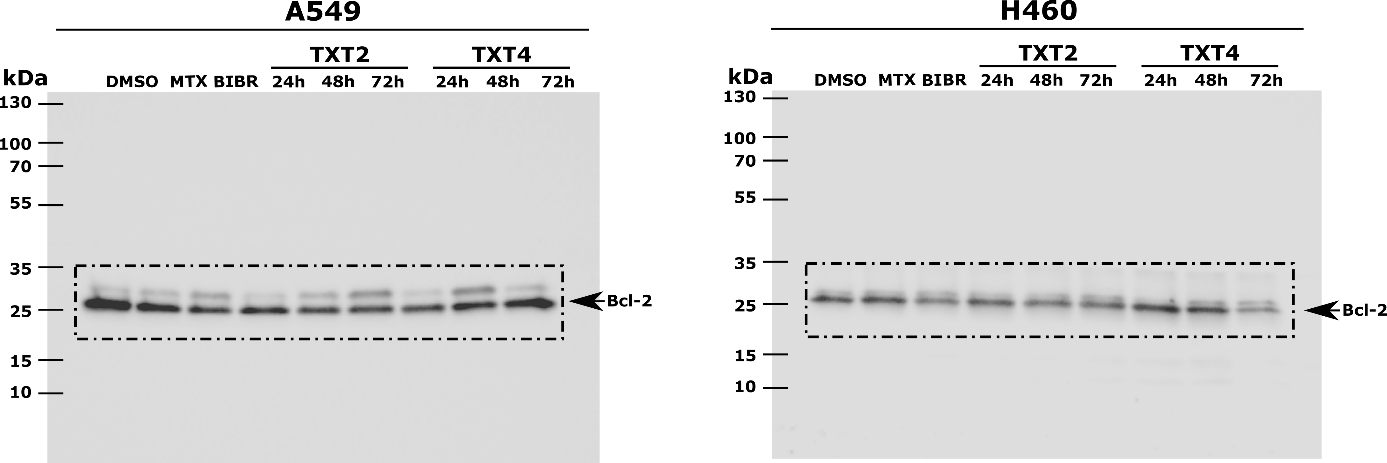


**Figure S26** Full-length blot presented in Figure 5 of the main article. The dotted box denotes the cropped regions of the blot.


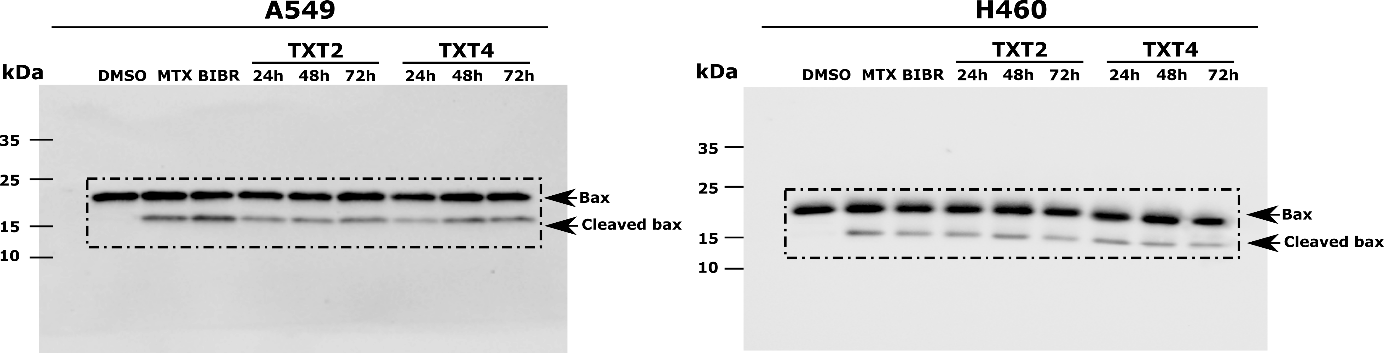


**Figure S27** Full-length blot presented in Figure 5 of the main article. The dotted box denotes the cropped regions of the blot.


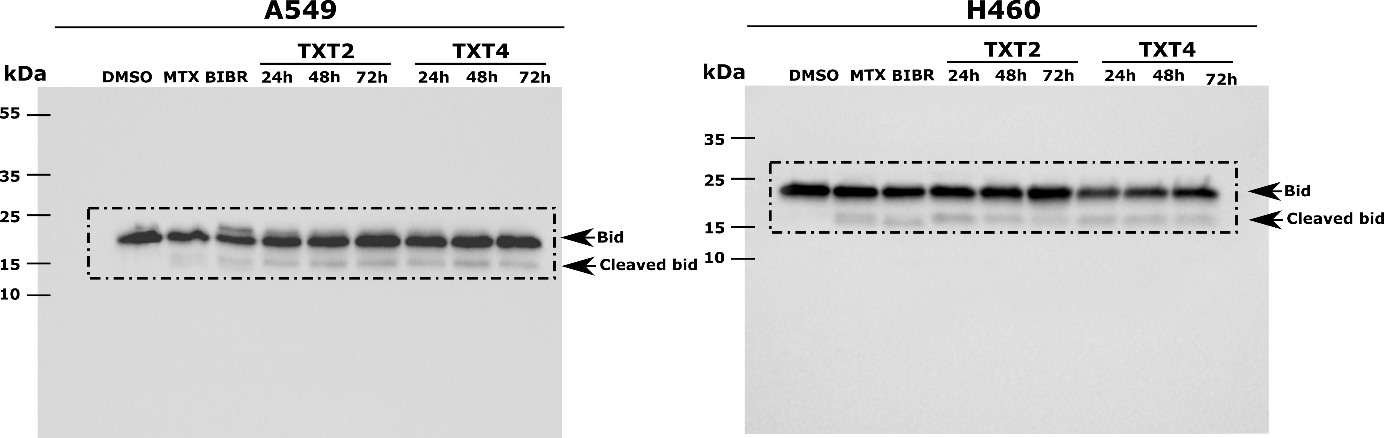


**Figure S28** Full-length blot presented in Figure 5 of the main article. The dotted box denotes the cropped regions of the blot.


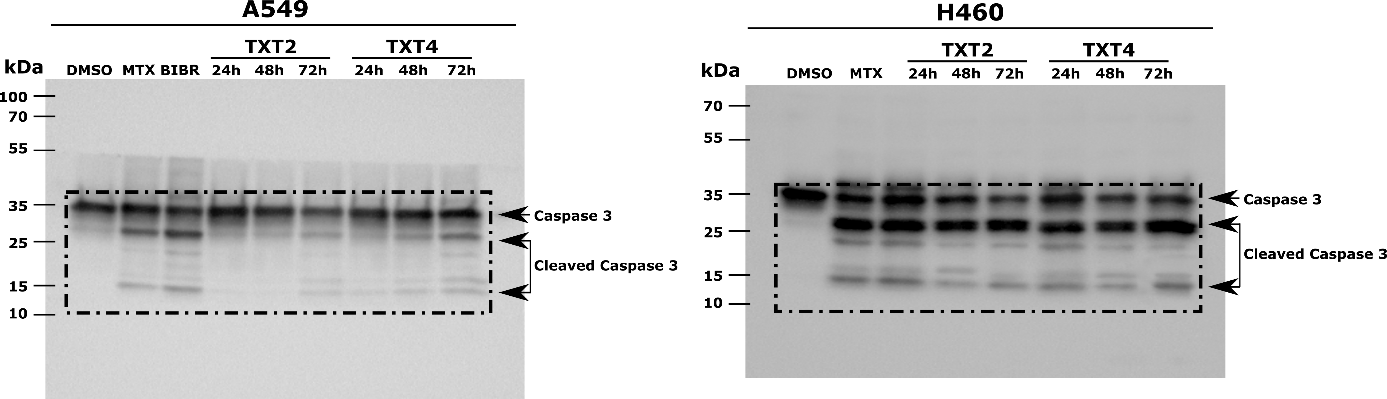


**Figure S29** Full-length blot presented in Figure 5 of the main article. The dotted box denotes the cropped regions of the blot.


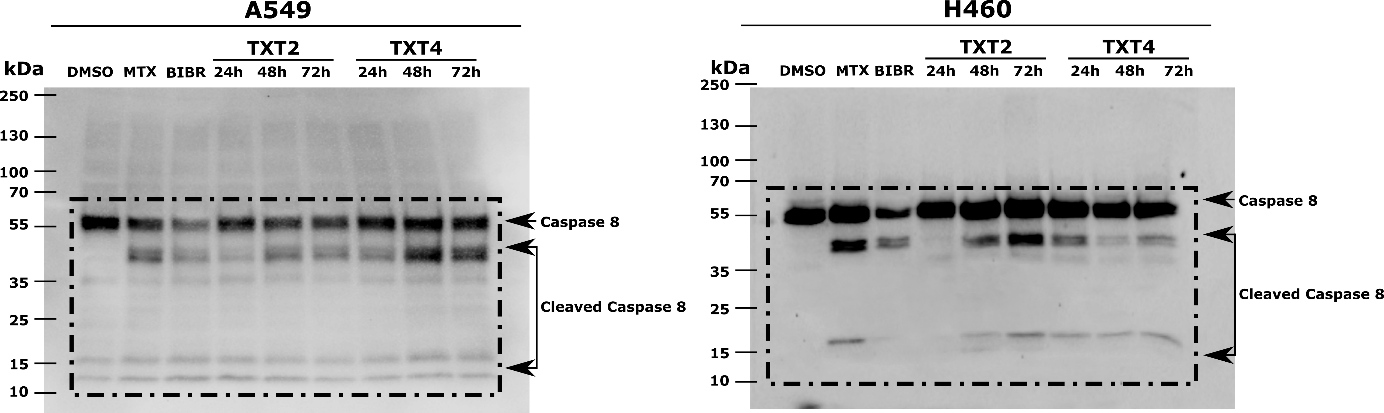


**Figure S30** Full-length blot presented in Figure 5 of the main article. The dotted box denotes the cropped regions of the blot.


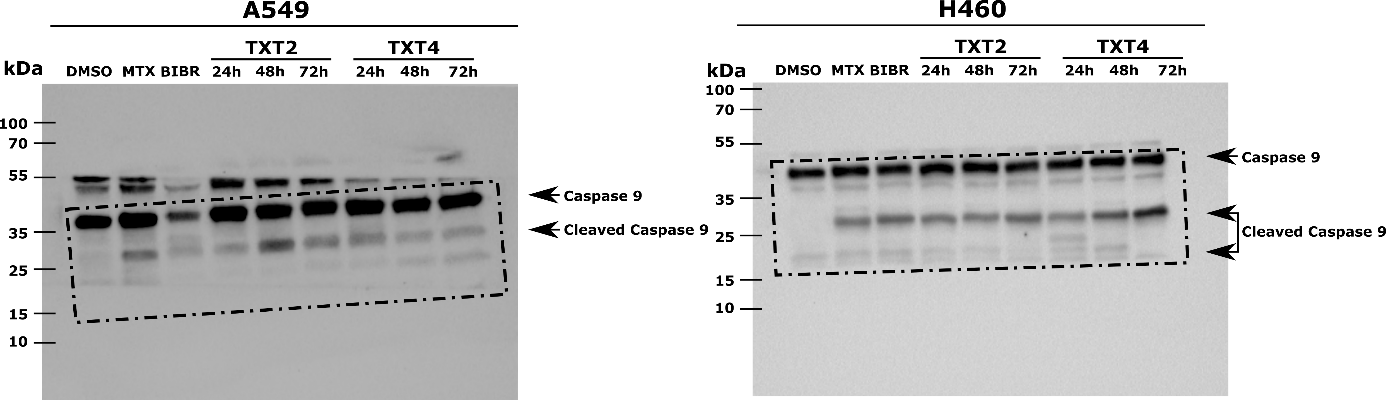


**Figure S31** Full-length blot presented in Figure 5 of the main article. The dotted box denotes the cropped regions of the blot.


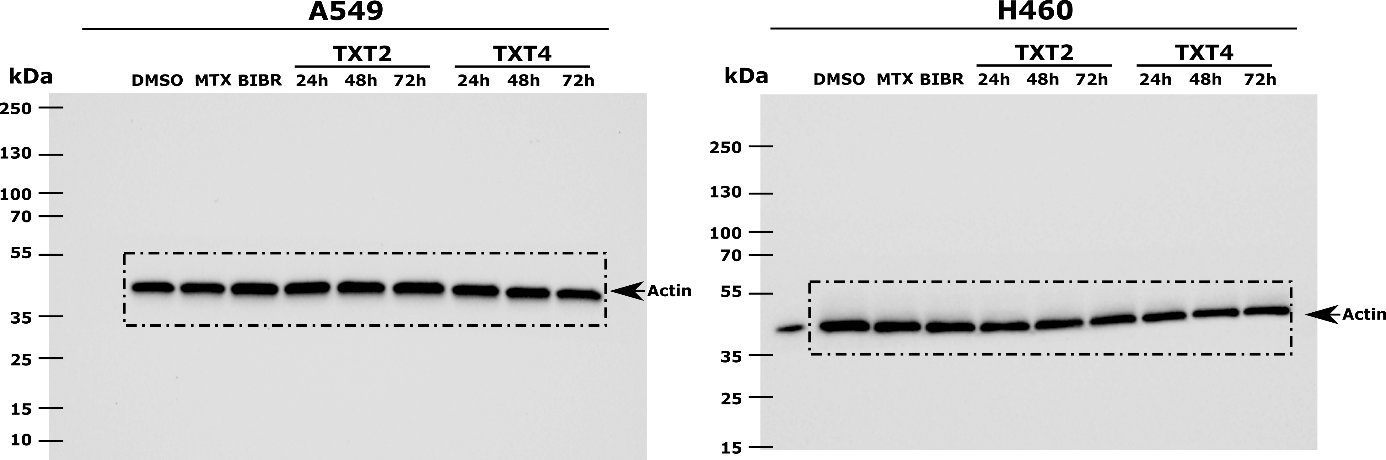


**Figure S32** Full-length blot presented in Figure 5 of the main article. The dotted box denotes the cropped regions of the blot.


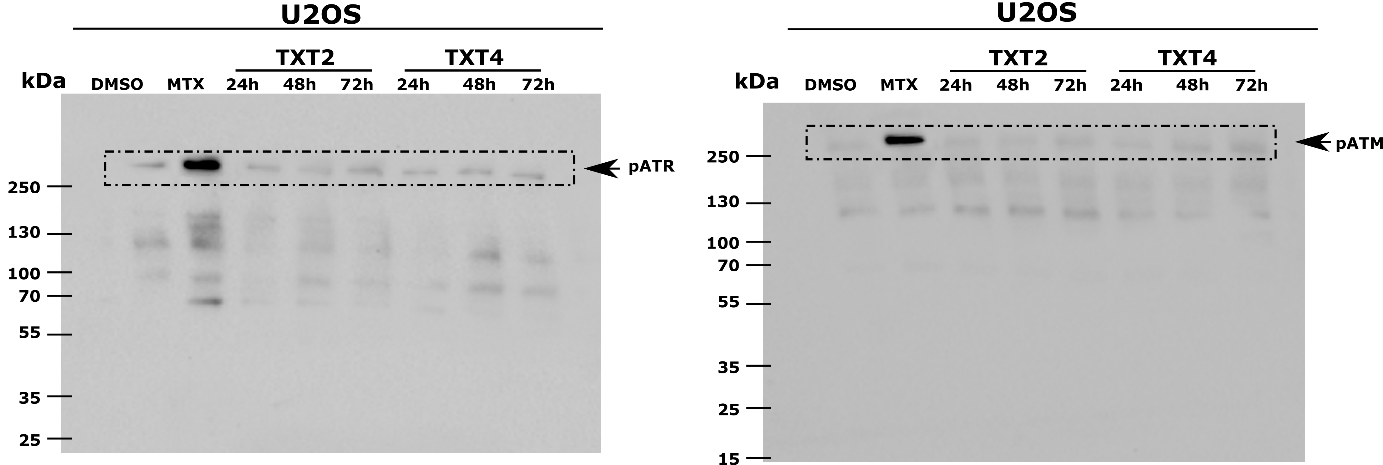


**Figure S*33*** Full-length blot presented in Figure S3 of the Supplementary Information. The dotted box denotes the cropped regions of the blot.


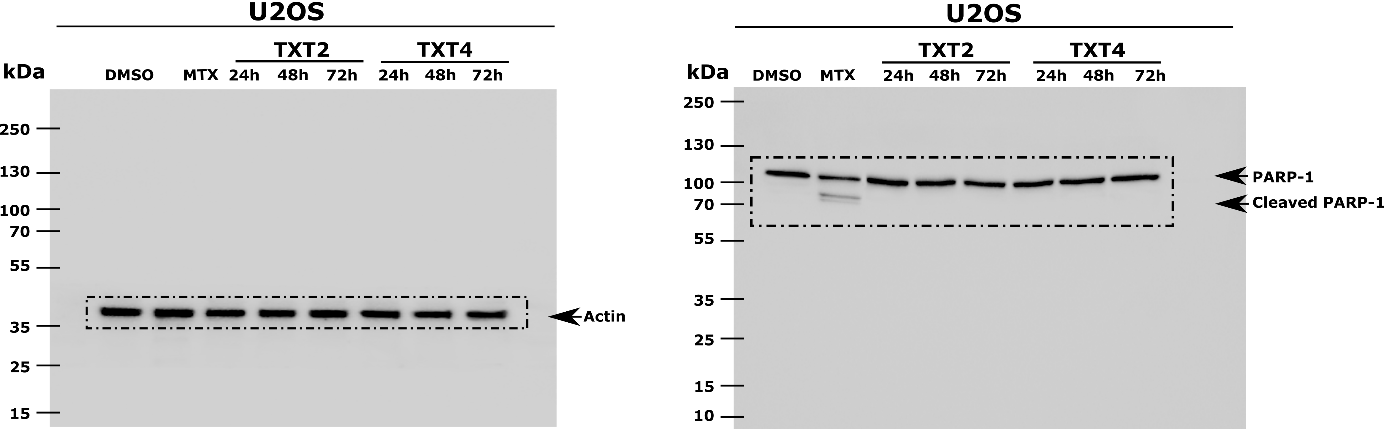


**Figure S*34*** Full-length blot presented in Figures S3 and S10 of the Supplementary Information. The dotted box denotes the cropped regions of the blot.


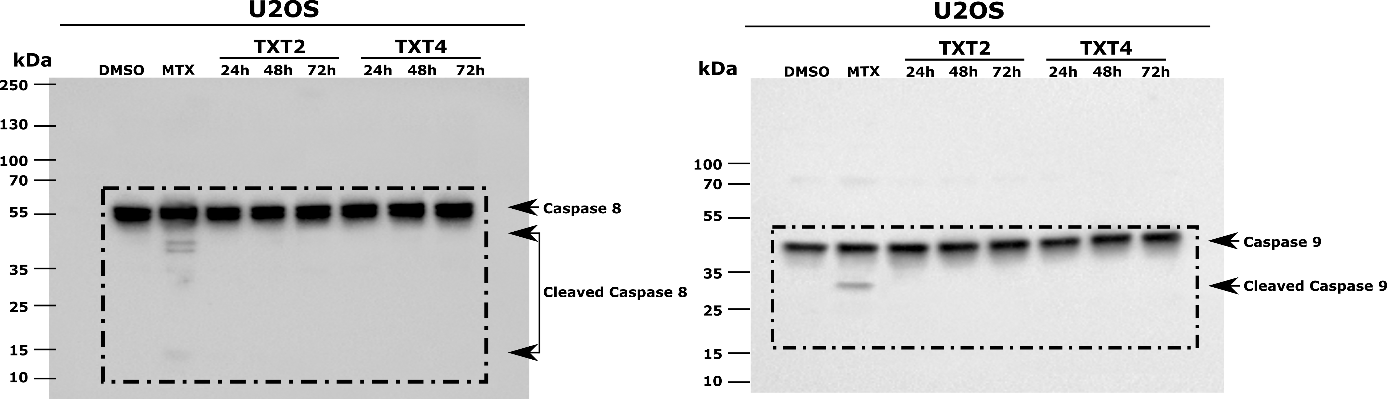


**Figure S*35*** Full-length blot presented in Figure S10 of the Supplementary Information. The dotted box denotes the cropped regions of the blot.


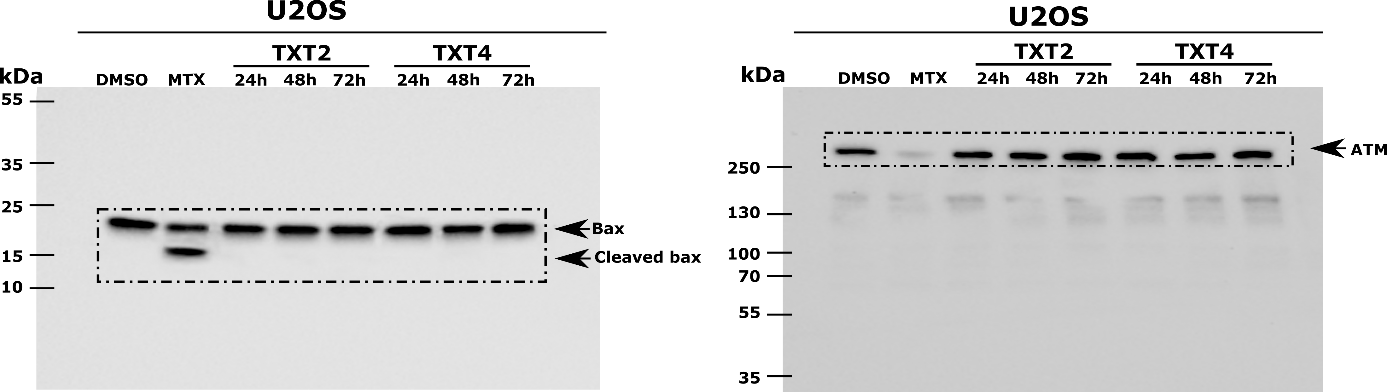


**Figure S*36*** Full-length blot presented in Figure S10 of the Supplementary Information. The dotted box denotes the cropped regions of the blot.


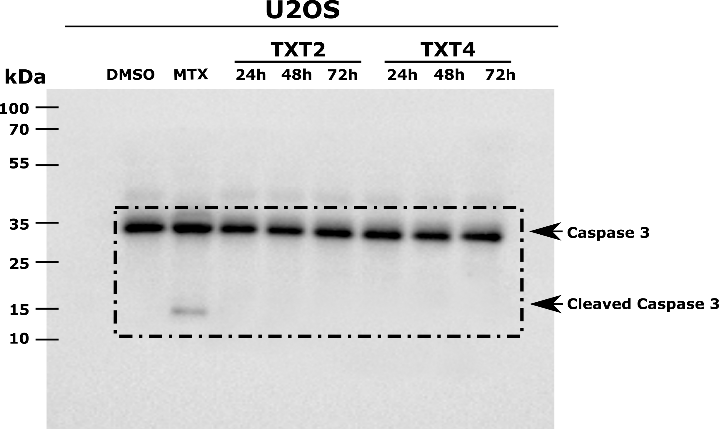


**Figure S*37*** Full-length blot presented in Figure S10 of the Supplementary Information. The dotted box denotes the cropped regions of the blot.
